# Hereditary chronic pancreatitis induced plasticity cooperates with mutant Kras in early pancreatic carcinogenesis

**DOI:** 10.1136/gutjnl-2025-335947

**Published:** 2025-12-19

**Authors:** Tanvi Vikrant Inamdar, Ferdinand Krannich, Nico Hesselbarth, Atul Verma, Teresa Vauti, Mariami Helena Jasaszwili, Ghanem El Kassem, Jasmine Hillmer, Tom Kaune, Michael Boettcher, Ivonne Regel, Heidi Griesmann, Irene Esposito, Markus Glaß, Monika Hämmerle, Patrick Michl, Helmut Laumen, Jonas Rosendahl

**Affiliations:** 1 Department of Internal Medicine I, University Medicine Halle, Martin Luther University Halle-Wittenberg, Halle (Saale), Germany; 2 Institute of Molecular Medicine, Section for Molecular Medicine of Signal Transduction, Faculty of Medicine, Martin Luther University Halle-Wittenberg, Halle (Saale), Germany; 3 Department of Medicine II, University Hospital, Ludwig Maximilian University of Munich, Munich, Germany; 4 Institute for Tumor Immunology, University Medicine Halle, Martin Luther University Halle-Wittenberg, Halle (Saale), Germany; 5 Institute of Pathology, Medical Faculty and University Hospital Düsseldorf, Heinrich-Heine-University, Düsseldorf, Germany; 6 Institute of Molecular Medicine, Martin Luther University Halle-Wittenberg, Halle (Saale), Germany; 7 Institute of Pathology, Martin Luther University Halle-Wittenberg, University Medical Center, Halle (Saale), Germany; 8 Dept. of Internal Medicine IV, Heidelberg University Hospital, Heidelberg, Germany

**Keywords:** Pancreatic Cancer, Pancreatitis, Pancreatic Disease, Pancreatic Damage

## Abstract

**Background:**

Chronic pancreatitis (CP) is a risk factor for pancreatic cancer, with inherited cases conferring a markedly increased risk. The underlying mechanisms driving malignant transformation by CP remain poorly understood.

**Objective:**

Combining a recently developed mouse model of CP carrying the human carboxypeptidase A1 (*CPA1*) p.N256K mutation with the established *Kras^G12D^* pancreatic cancer model, we characterised mechanisms linking chronic inflammation to early pancreatic carcinogenesis.

**Design:**

We crossed *Cpa1*^*N256K*^ mice (Cpa1) with *Ptf1a^Cre^;Kras^LSL-G12D^* (KC). In Cre, Cpa1, KC and KC-Cpa1 mice, we performed phenotypical characterisation at five early time points and in an ageing cohort. Assessment of histology combined with both RNA-sequencing and single-cell RNA-sequencing was performed to analyse metaplasia, preneoplastic lesions and cellular heterogeneity.

**Results:**

KC-Cpa1 pancreata displayed a stark increase in remodelling, fibrosis and formation of metaplastic lesions as compared with KC. *Cpa1^N256K^* induced extensive plasticity in both the acinar and ductal compartment, including an early acinar-to-ductal metaplasia state in acinar cells characterised by an upregulation of endoplasmic reticulum stress markers and an inflammatory ductal phenotype (iDucts). We characterised the complex cell-cell communication networks underlying both pancreatic inflammation and early carcinogenesis, revealing disease-specific signalling between ductal cells, granulocytes and fibroblasts.

**Conclusions:**

The humanised KC-Cpa1 mouse model reveals the interplay of inflammation in hereditary CP and carcinogenesis. *Cpa1^N256K^*-induced plasticity in acinar and ductal cells, inflammation and cell-cell interaction networks cooperate with *Kras^G12D^* in early pancreatic carcinogenesis.

**WHAT IS ALREADY KNOWN ON THIS TOPIC**

- Chronic pancreatitis (CP) is a significant risk factor for pancreatic cancer.
- Mouse models carrying the *Kras^G12D^* mutation recapitulate pancreatic carcinogenesis.
- The hereditary CP mutation *Cpa1^N256K^* leads to spontaneous CP in a humanised mouse model.

**WHAT THIS STUDY ADDS**

- Combining *Cpa1^N256K^* and *Kras^G12D^* in a humanised mouse model, we characterised the interplay of hereditary CP and pancreatic carcinogenesis.
- We identified distinct populations of *Kras^G12D^*-induced and *Cpa1^N256K^*-induced acinar-to-ductal metaplasia and uncovered an inflammatory ductal phenotype (iDucts) unique to CP.
- We mapped intercellular signalling networks involving acinar and metaplastic cells, granulocytes, fibroblasts and iDucts, revealing synergistic interactions between inflammatory and oncogenic transcriptional programmes.

**HOW THIS STUDY MIGHT AFFECT RESEARCH, PRACTICE, OR POLICY**

- This mouse model complements the well-established caerulein-induced models by developing spontaneous CP, offering a more physiologically relevant system to study CP as a risk factor for pancreatic cancer development.
- This model may support the development of improved diagnostic, preventive and therapeutic strategies targeting inflammation-driven carcinogenesis.

## Introduction

 Chronic pancreatitis (CP) is a long-standing inflammatory disease often leading to exocrine and endocrine insufficiency.^1 2^ Although alcohol misuse remains the most common aetiology, numerous genetic associations have been identified over the past several decades.^3^,^10^ These genetic variants contribute to disease development through three major pathways: trypsin-dependent, misfolding-dependent and ductal.^11^ The sequelae of CP are diverse with a notably increased risk for pancreatic cancer that is up to 69-fold higher in patients with inherited disease.^12^,^14^ The mechanisms driving the malignant transformation from pancreatitis to pancreatic cancer remain poorly understood.

In light of growing knowledge of genetic alterations predisposing to CP, animal models exhibiting a spontaneous disease phenotype have been developed in recent years.^15^,^24^ A mouse model carrying a knock-in of the human p.N256K carboxypeptidase A1 (*CPA1*) mutation induced endoplasmic reticulum (ER) stress and led to CP over time.^21^ Histological analysis of this model revealed features of acinar-to-ductal metaplasia (ADM) with pseudotubular complexes, a hallmark of pancreatic cancer development.^25^ Moreover, deleterious mutations in *CPA1* and carboxypeptidase B1 (*CPB1*) were enriched in a combined set of sporadic and familial cases of pancreatic cancer, strengthening the link between CP and pancreatic cancer development.^26^

Pancreatic ductal adenocarcinoma (PDAC) is a highly lethal malignancy with a dismal 5-year survival rate of 13%.^27 28^ Activating point mutations in the *KRAS* gene are found in approximately 88% of human PDAC cases,^29^ with the glycine-to-aspartate mutation at position 12 (G12D) being particularly prevalent and associated with a worse prognosis as compared with the other mutations of *KRAS*.^30^ This mutation has been effectively modelled in *Ptf1a^Cre^;Kras^LSL-G12D^* (KC) mice, which recapitulate the full spectrum of PDAC progression.^31^ However, oncogenic expression of *Kras* alone leads to a relatively low incidence of PDAC, as tumour development typically requires additional genetic mutations, such as loss of *TP53*, *CDKN2A* and *SMAD4* (SMAD family member 4).^32^ Alternatively, animal models harbouring *Kras* mutations can develop the full spectrum of precursor lesions and PDAC when CP is experimentally induced by caerulein.^33^

Although early detection of pancreatic cancer has the potential to greatly expand therapeutic opportunities, it remains a significant challenge due to the disease’s low prevalence and the absence of reliable screening modalities.^34^ In-depth characterisation of early molecular events in at-risk populations may provide a critical window for timely intervention to improve outcome.^35^ One such scenario is CP, marked by a persistent inflammatory microenvironment that creates a permissive niche for the initiation and progression of PDAC. In this study, we used the spontaneous *Cpa1*-driven CP model in combination with oncogenic *Kras* to investigate inflammation-driven mechanisms underlying PDAC initiation.

## Materials and methods

### Mice

Animal experiments were in accordance with institutional guidelines and approved by the Landesverwaltungsamt Saxony-Anhalt (MLU-K6IM13;-K6IM14;-K6IM15;-K6IM16;-K6IM17). The mouse strains Cre (*Ptf1a^+/Cre^*), Cpa1 (*Cpa1^N256K/N256K^*), KC (*Ptf1a^+/Cre^Kras^LSLG12D/+^*) and KC-Cpa1 (*Ptf1a^+/Cre^Kras^LSLG12D/+^Cpa1*^*N256K/N256K*^) were housed in pathogen-free facilities in groups of three to five mice at 22±2°C on 12 hours light/dark cycles with ad libitum access to water and food (standard chow diet; Altromin maintenance-diet 1324SP, breeding-diet 1314SP) and sacrificed at defined ages as indicated. The ageing cohort was euthanised on reaching humane endpoints. Generation of the new KC-Cpa1 mouse strain, genotyping primers, phenotypical characterisation, RNA-seq analysis and representative results are described in the online supplemental material.

### Tissue dissociation for single cell sequencing

Mice were anaesthetised with isoflurane (Vetflurane, Virbac) followed by cardiac perfusion using ice-cold 1X phosphate-buffered saline (PBS), the pancreas was excised, cut into small pieces in digestion medium containing collagenase P (Roche, 11213873001), soybean trypsin-inhibitor (Gibco, 17075–029), 1% BSA (SERVA, 11930.04) and DNase I (Sigma-Aldrich, D5025), and incubated 20–40 min at 37°C on MACSmix^TM^ tube rotator (Miltenyi Biotec). For fibrotic tissue, prolonged digestion was performed. Digested samples were quenched with RPMI medium (Gibco, 21875–034) supplemented with 10% fetal calf serum (FCS), centrifuged at 350 rcf for 5 min at 4°C, the cell pellets washed with Hanks Balanced Salt solution (HBSS, Gibco, 14175–095) supplemented with soybean trypsin-inhibitor and DNase I, passed through a 70 µm filter, pelleted and resuspended in HBSS supplemented with soybean trypsin-inhibitor, DNase I and Y-27632 dihydrochloride (Sigma-Aldrich, Y-0503) and kept on ice until further processing.

### Single-cell sequencing library preparation

Library preparation was performed using Chromium Next GEM Single Cell 3ʹ Reagent Kits V.3.1 (10x Genomics, PN-1000268) following manufacturer’s protocol. Cells were counted and cell numbers adjusted in HBSS supplemented with 1% BSA (bovine serum albumin). 10 000 Cre or Cpa1 and 7000 KC or KC-Cpa1 cells were loaded on Chromium (10x Genomics) device. Sequencing was performed on Illumina NovaSeq X Plus Series (PE150) by Novogene (Munich, Germany).

### Single-cell analysis

Following the alignment of FASTQ files, raw count matrices were merged and preprocessed using Seurat^36^ (V.5.0.3), including quality control, doublet detection, normalisation, dimensionality reduction and clustering. Clusters were annotated based on our annotation strategy (online supplemental table 1). Pseudotime trajectories were inferred using Monocle3^37^ (V.1.3.4). Pseudobulk profiles of individual cell types were calculated based on normalised gene expression across the entire measured transcriptome. Cell-cell interactions were predicted using CellChat^38^ (V.2.1.2). Gene set enrichment analysis (GSEA) was performed using Seurat’s implementation of the enrichR^39^ (V.3.2) database and clusterProfiler^40^ (V.4.10.1).

For detailed methods, see online supplemental materials.

## Results

### *Cpa1*^*N256K*^ increases pancreas remodelling in early pancreatic carcinogenesis

**Figure 1:**
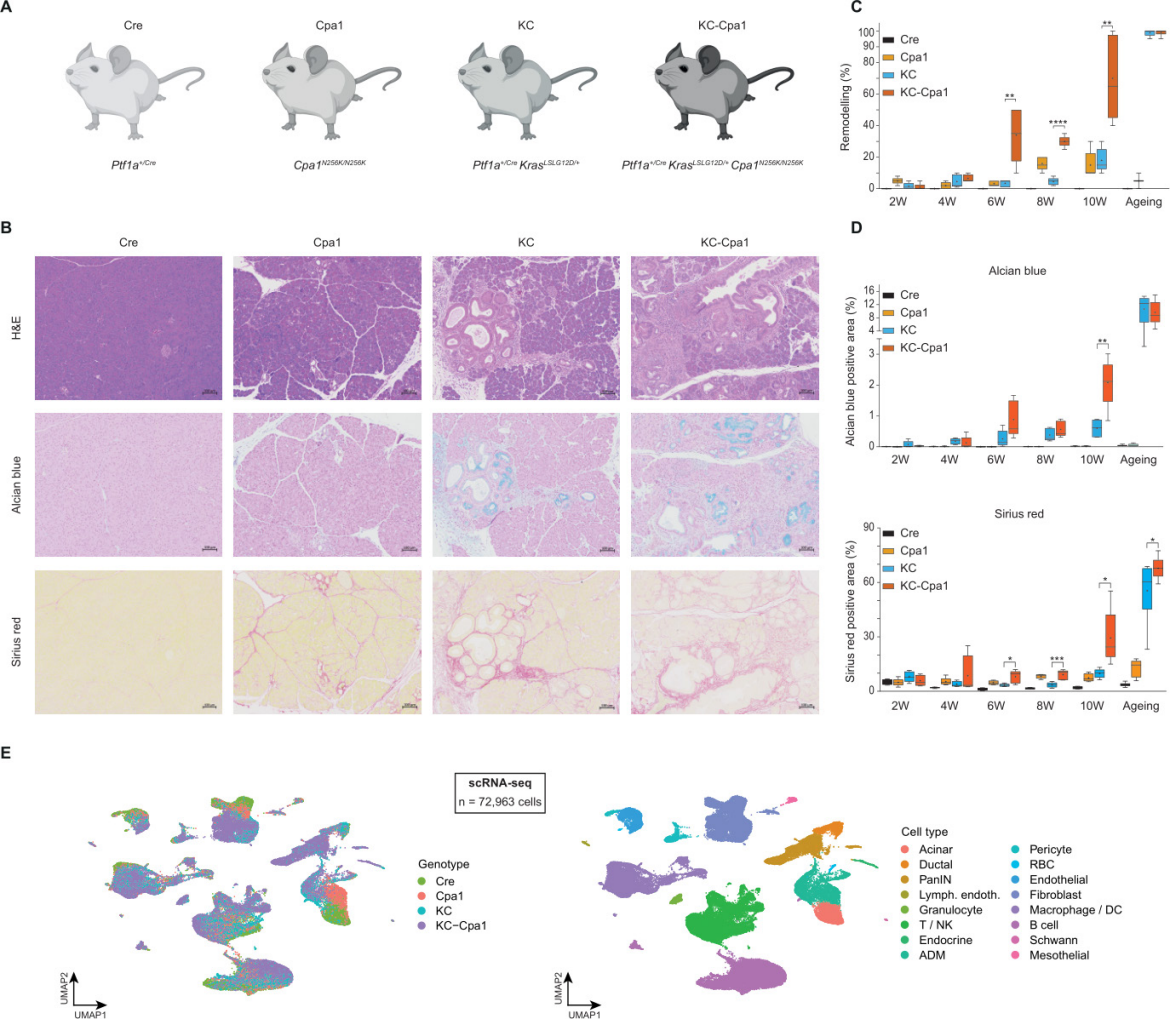
Capturing the interplay of pancreatic inflammation and carcinogenesis through the mouse model KC-Cpa1. (**A**) Genetically engineered mouse models analysed in this study. (**B**) Representative H&E, Alcian blue and Sirius red stainings of Cre, Cpa1, KC and KC-Cpa1 pancreata at the age of 8 weeks. (**C, D**) Quantification of remodelling in Cre, Cpa1, KC and KC-Cpa1 mice through pathological assessment (**C**) and image analysis of stained areas (**D**). (**E**) UMAP (uniform manifold approximation and projection) plots depicting genotype and cell type composition of scRNA-seq (single-cell RNA-sequencing) samples. ADM, acinar-to-ductal metaplasia; DC, dendritic cell; Lymph endoth, lympathic endothelial; NK, natural killer cell; PanIN, pancreatic intraepithelial neoplasia; RBC, red blood cell.

To unravel the impact of CP induced by *CPA1^N256K^* on pancreatic homoeostasis and compare metaplasia in CP to early stages of pancreatic carcinogenesis, we leveraged the recently published humanised CP knock-in mouse model *Cpa1^N256K^* (Cpa1)^21^ and the well-established pancreatic cancer mouse model *Ptf1a^Cre^;Kras^LSL-G12D^* (KC)^31^ (figure 1A). To further assess the direct impact of *Cpa1^N256K^*-induced CP on early pancreatic carcinogenesis, we crossed both strains resulting in the *Ptf1a^Cre^;Kras^LSL-G12D^;Cpa1^N256K^* (KC*-*Cpa1) mouse strain.

Histology of Cre (*Ptf1a^Cre^*), Cpa1, KC and KC-Cpa1 pancreata was evaluated at six ages through both pathological assessment and image slide quantification (figure 1B–D*,* onlinesupplemental figures 1^3^). Cpa1 pancreata displayed diffuse ADM lesions and only negligible pancreatic intraepithelial neoplasia (PanIN) formation. Starting at the age of 8 weeks, Cpa1 pancreata developed fibrosis. In KC*-*Cpa1 mice, we found a stark increase in remodelling compared with KC (figure 1C). As such, KC-Cpa1 pancreata were characterised by the rapid formation of ADM and PanIN lesions. These findings were corroborated by a significant increase of Alcian blue and Sirius red (figure 1D), and enhanced Muc5ac (online supplemental figure 4A and B) and α-SMA (alpha smooth muscle actin) (online supplemental figure 4C and D) staining in KC-Cpa1. At the age of 10 weeks, 6 out of 10 KC-Cpa1 animals had developed PDAC, in contrast to 1 out of 10 KC animals (online supplemental table 2).

Previous studies have characterised a remarkable heterogeneity of epithelial and immune cell types in both pancreatitis^41^ and early pancreatic carcinogenesis.^42 43^ To characterise cellular heterogeneity, signalling processes and plasticity underlying *Cpa1^N256K^*-induced CP and carcinogenesis, we performed both RNA-sequencing (RNA-seq) and single-cell RNA-sequencing (scRNA-seq). RNA-seq was performed in a cohort capturing five early time points and a late ageing time point (online supplemental figure 5), (onlinesupplemental tables 3  4). scRNA-seq was performed in all genotypes at 8 weeks of age, with additional analyses performed for KC and KC-Cpa1 at later time points (figure 1E). This yielded a total of 72 963 cells included in the analysis, with 16 main cell clusters identified and annotated (figure 1E, online supplemental table 1).

### *Cpa1*^*N256K*^ induces an early ADM state in acinar cells

**Figure 2:**
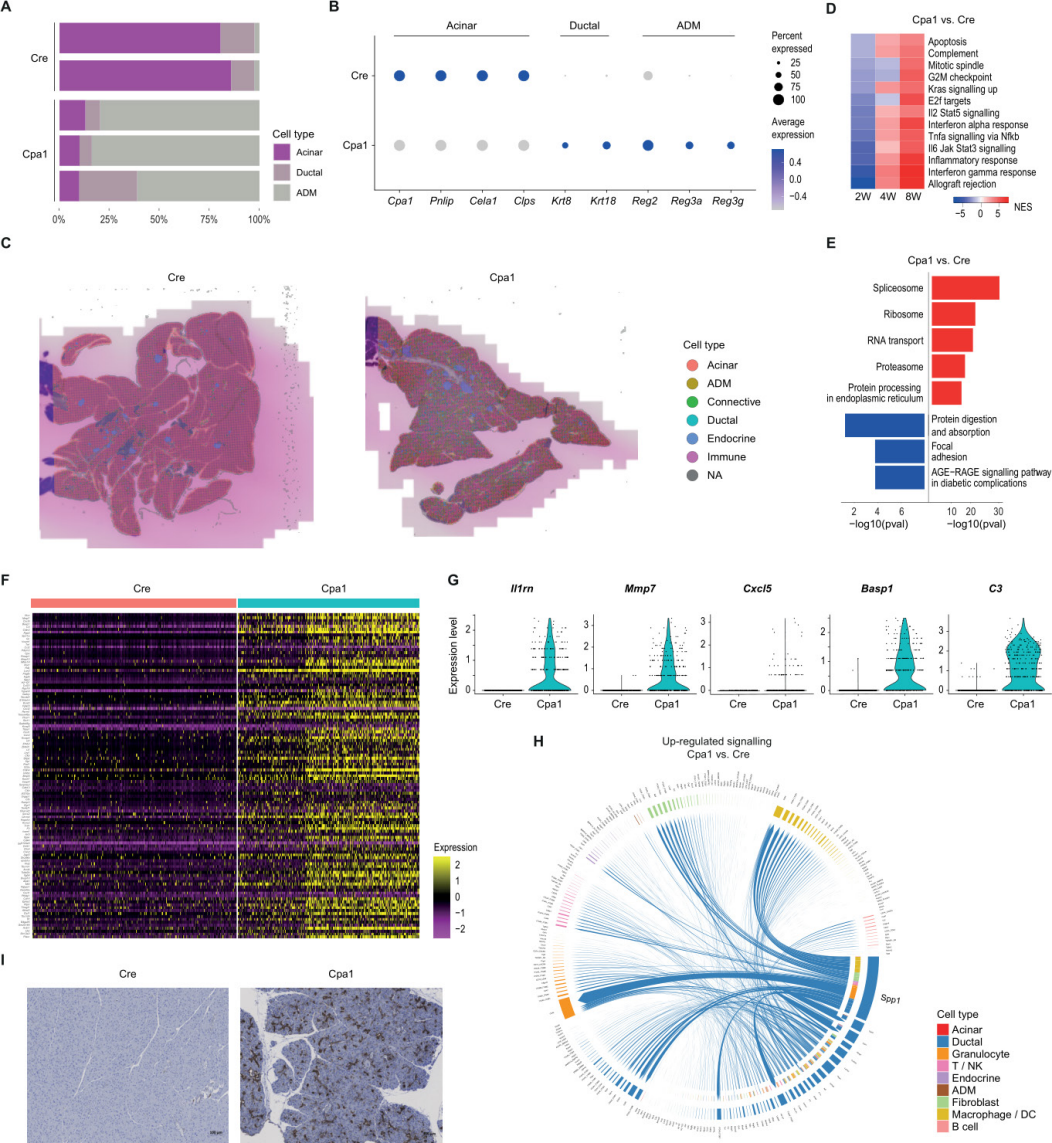
Plasticity characterises the exocrine compartment of Cpa1 mice. (**A**) Cell type composition of the epithelial compartment in 8-week-old Cre (*Ptf1a^+/Cre^*) and Cpa1 (*Cpa1^N256K/N256K^*) mice. Each bar corresponds to a biological replicate. (**B**) Dot plot depicting acinar, ductal and ADM marker expression in the acinar compartment (acinar and ADM clusters) of 8-week-old Cre and Cpa1 mice. (**C**) Spatial deconvolution of Cre and Cpa1 pancreata, based on single-cell data of Cre and Cpa1 mice at corresponding ages. (**D**) Gene set enrichment RNA-seq of Hallmark pathways in pancreata of 2-week-old, 4-week-old and 8-week-old Cpa1 mice compared with Cre. (**E**) Gene set enrichment scRNA-seq of KEGG (Kyoto encyclopedia of genes and genomes) pathways in the acinar compartment of 8-week-old Cpa1 mice compared with Cre. (**F**) Heatmap depicting ductal cells in 8-week-old Cre and Cpa1 mice. Expression of markers upregulated in Cpa1 compared with Cre mice. (**G**) Violin plots visualising differentially expressed marker genes of iDucts (inflammatory ducts) in 8-week-old Cpa1 mice compared with Cre. (**H**) Chord diagram visualising the upregulated signalling in ductal cells of 8-week-old Cpa1 mice compared with Cre. (**I**) iDucts marker Basp1 staining of 8-week-old Cpa1 and Cre mice. ADM, acinar-to-ductal metaplasia; DC, dendritic cell; NA, not applicable.

ADM is a hallmark of both CP and early pancreatic carcinogenesis. It is thought to serve as a tissue-protective programme on inflammatory stimuli,^44^ being characterised by an upregulation of ductal markers (eg, *Krt19*, *Sox9*, *Cftr*), downregulation of digestive enzymes (eg, *Cpa1*, *Pnlip*, *Cela2*) and expression of ADM markers (eg, *Reg3a*, *Reg3g*).^45^ To study CP-induced acinar plasticity, we turned to the acinar compartment of Cpa1 mice. Here, the majority of acinar cells exhibited a transition to ADM (figure 2A). Although downregulated, acinar cells in Cpa1 still displayed pronounced digestive enzyme expression, in line with an early ADM state (figure 2B). To exclude potential scRNA-seq artefacts induced by the cell isolation process, we further performed sequencing-based spatial transcriptomics and spatial deconvolution of Cpa1 and Cre pancreata. In line with histology (figure 1B, online supplemental figure 1B) and Krt19 staining (online supplemental figure 4E), we found Cpa1 pancreata to be characterised by diffuse ADM lesions spread across the tissue (figure 2C, online supplemental figure 6B). Tissue domains of Cpa1 and Cre pancreata showed only little overlap (online supplemental figure 6C), underscoring the distinct spatial architecture induced by *Cpa1^N256K^*.

We then sought to understand the molecular underpinnings of the *Cpa1^N256K^*-induced early ADM state in acinar cells. For *Cpa1^N256K^*, an impaired secretion and intracellular accumulation of heterologously expressed protein in 293 T cells has been reported. Consequently, Cpa1 mice exhibited ER stress with an upregulation of the ER stress markers *DDIT3*, *HSPA5* and *XBP1* splicing in the total pancreas.^21^ We found retroviral overexpression of *Cpa1^N256K^* in the acinar cell line 266–6 to induce an upregulation of ER chaperones such as *Hspa5, Sdf2l1* and *Hsp90b1* in RNA-seq, with significant enrichment of the ER chaperone complex (online supplemental table 5). GSEA of pancreata and the acinar compartments of Cpa1 versus Cre mice confirmed upregulation of ER stress-related pathways and inflammation-related pathways (figure 2D and E). Interestingly, expression levels of ER stress markers correlated with the degree of acinar cell dedifferentiation in single-cell data, with higher ER stress marker expression corresponding to the most pronounced downregulation of acinar markers (online supplemental figure 6A).

### Ductal cells display an inflammatory phenotype in Cpa1 mice

In light of recently described plasticity in the ductal compartment with potential roles of ductal subtypes in pancreas regeneration, inflammation and pathogenesis,^46 47^ we then analysed the impact of *Cpa1^N256K^* on ductal cells in Cpa1 mice. Notably, we identified two subclusters of ductal cells in Cpa1, one largely reflecting the expression patterns of Cre ductal cells and one clustering differently from Cre ductal cells (figure 2F*,* online supplemental figure 7A and B). In Cpa1 ductal cells, we found inflammation-related pathways to be upregulated, including cytokine-cytokine receptor interaction and *NF-κB* (nuclear factor kappa-light-chain-enhancer of activated B cells) signalling (online supplemental figure 7C). Cpa1 ductal cells displayed pronounced upregulation of inflammation-related genes (eg, *Il1rn*, *C3, Cxcl5*) and matrix metalloproteinases (eg, *Mmp7*) (figure 2G, online supplemental table 6). In analogy to inflammatory cancer-associated fibroblasts (iCAFs), we termed this subtype of ductal cells ‘iDucts’ (inflammatory ducts).

To understand the role of iDucts in *Cpa1^N256K^*-induced CP, we then inferred cell-cell communication networks and compared signalling in the ductal compartment of Cpa1 to Cre. Here, we found a stark increase of signalling, particularly in the interaction of ductal cells with immune cells and fibroblasts (figure 2H). Secreted phosphoprotein 1 (*Spp1*) signalling was particularly upregulated in iDucts, previously characterised in pancreatic ductal cells.^47^ IHC (immunohistochemistry) staining of Basp1 revealed iDucts to be spread across Cpa1 tissue, and negative staining of Cre ductal cells (figure 2I, online supplemental figure 7D).

### Oncogenic *Kras* primes ADM towards preneoplasia

**Figure 3:**
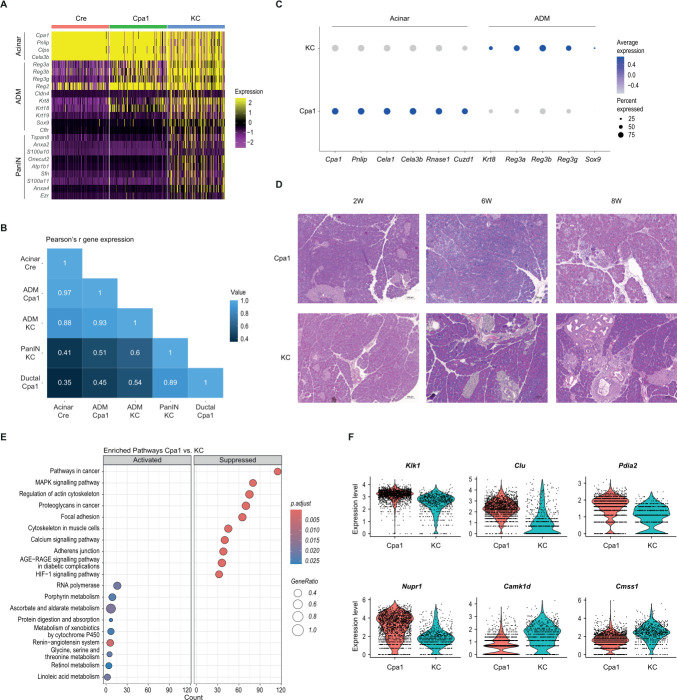
Metaplasia in inflammation and early carcinogenesis. (**A**) Heatmap depicting acinar, ADM (acinar-to-ductal metaplasia) and PanIN (pancreatic intraepithelial neoplasia) marker expression in 8-week-old Cre (*Ptf1a^+/Cre^*), Cpa1 (*Cpa1^N256K/N256K^*) and KC (*Ptf1a^+/Cre^Kras^LSLG12D/+^*) mice. (**B**) Heatmap depicting global correlation of gene expression across different cell populations in 8-week-old Cre, Cpa1 and KC mice. (**C**) Dot plot visualising acinar, ADM and ductal marker expression in the ADM clusters of 8-week-old KC and Cpa1 mice. (**D**) Representative H&E stainings of Cpa1 and KC pancreata at different ages. (**E**) Gene set enrichment of KEGG (Kyoto encyclopedia of genes and genomes) pathways in ADM of 8-week-old Cpa1 mice compared with KC. (**F**) Violin plots depicting marker gene expression in the ADM clusters of 8-week-old Cpa1 vs KC mice.

In contrast to the early ADM in Cpa1, dedifferentiation in KC reached as far as PanIN, the most common preneoplastic lesion to pancreatic cancer (figure 3A, onlinesupplemental figures 1^4^). Thus, ADM in KC spanned a broader spectrum of stages, reaching back as far as late ADM on the verge of PanIN formation. Global correlation analysis of gene expression levels further supported this observation, with Cre acinar cells displaying a stronger correlation with the Cpa1 ADM cluster (r=0.97) than with KC ADM (r=0.88) (figure 3B). Consistent with these findings, ADM in KC mice was characterised by an even more pronounced downregulation of acinar markers (eg, *Cpa1, Pnlip, Cela3b*) and upregulation of ductal markers (eg, *Krt8, Sox9*) compared with Cpa1 (figure 3C). In RNA-seq of the ageing cohort (online supplemental table 4), we found a complete downregulation of digestive enzymes and pronounced upregulation of PanIN markers (eg, *Muc5ac*, *Gkn1/2*) in KC animals, while Cpa1 pancreata remained characterised by ADM marker expression (eg, *Cldn4*, *Reg3g*).

We then sought to identify genes and pathways discriminating the *Cpa1^N256K^*-induced inflammatory ADM from the ADM in carcinogenesis (figure 3D), thus offering insights into molecular mechanisms priming the oncogenic ADM towards preneoplasia and ultimately cancer. Pathways related to carcinogenesis were upregulated in KC ADM, including MAPK (mitogen-activated protein kinase), calcium and HIF-1 (hypoxia inducible factor 1) signalling (figure 3E). We also found several pathways related to cytoskeleton and extracellular matrix (ECM) to be upregulated in KC. In Cpa1, we found several metabolic pathways to be upregulated, in contrast to the recently described metabolic reprogramming of cells undergoing *Kras^G12D^*-induced ADM.^48^ Top upregulated genes in Cpa1 included secreted *Clu* (clusterin), an apoptosis inhibiting gene,^49^ and genes with chaperone activities in the pancreas such as *Pdia2* (protein disulfide isomerase associated 2),^50^ in line with ER stress and UPR (unfolded protein response) due to misfolded *Cpa1^N256K^* (figure 3F).

### Acinar-intrinsic transcriptional programmes accelerate dedifferentiation in KC-Cpa1

**Figure 4:**
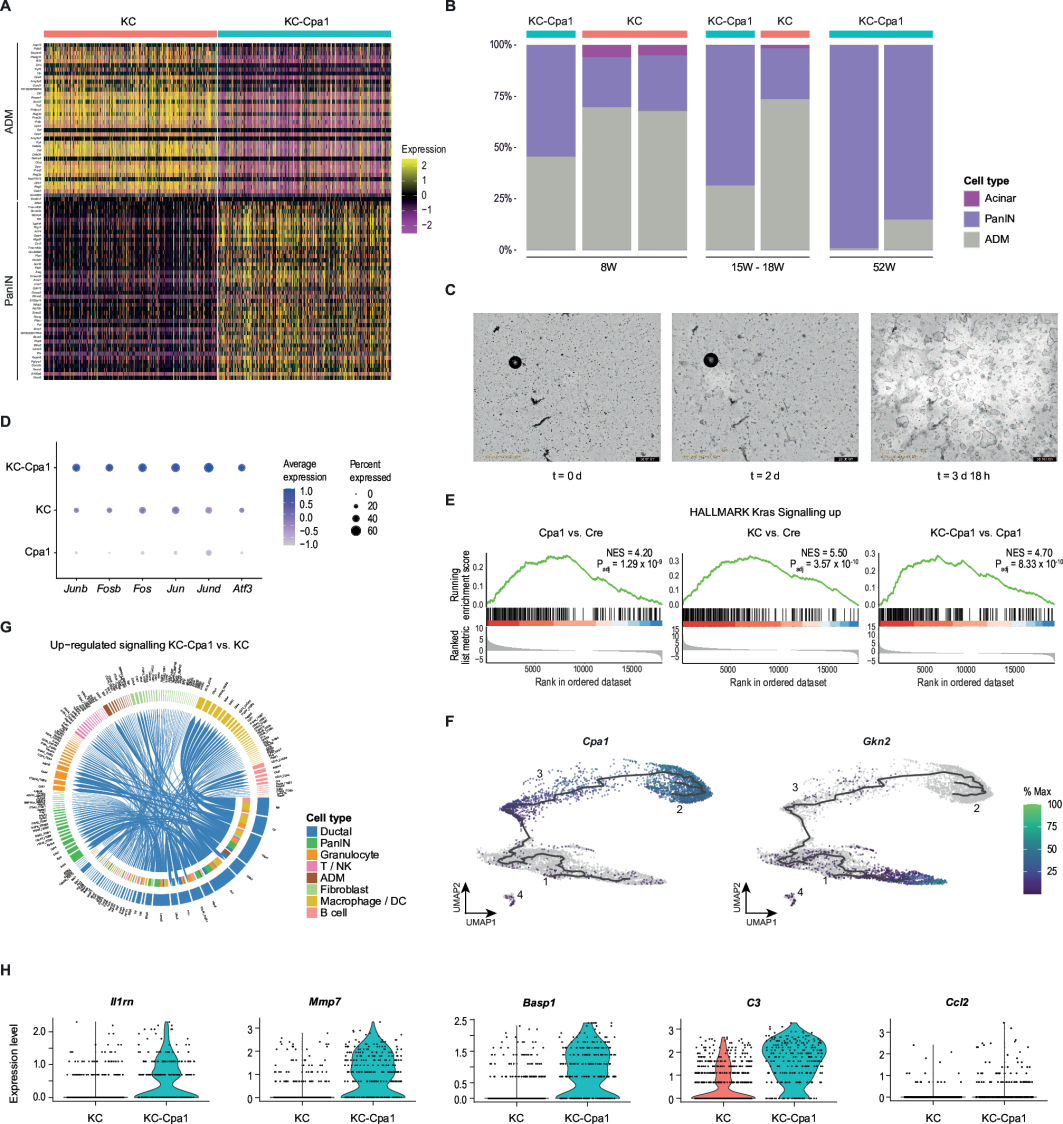
Interplay of pancreatic inflammation and early carcinogenesis in KC-Cpa1. (**A**) Heatmap depicting ADM (acinar-to-ductal metaplasia) and PanIN (pancreatic intraepithelial neoplasia) marker expression in 8-week-old KC (*Ptf1a^+/Cre^Kras^LSLG12D/+^*) and KC-Cpa1 (*Ptf1a^+/Cre^Kras^LSLG12D/+^Cpa1*^N256K/N256K^) mice. (**B**) Cell type composition of acinar and metaplastic cells in KC and KC-Cpa1 mice across time points. Each bar corresponds to a biological replicate. (**C**) Live-cell imaging of KC-Cpa1 acini embedded in collagen, and incubated with TGF-α and caerulein. (**D**) Dot plot visualising AP-1 complex transcription factor expression in 8-week-old KC-Cpa1, KC and Cpa1 (*Cpa1^N256K/N256K^*) mice. (**E**) Gene set enrichment of *Kras* signalling in RNA-seq of 8-week-old Cre (*Ptf1a^+/Cre^*), Cpa1, KC and KC-Cpa1 mice. (**F**) UMAP plots depicting pseudotime trajectory inference through ADM (*3*) and PanIN (*1*,*4*) clusters of KC-Cpa1 samples at the age of 8 weeks, 18 weeks and 52 weeks. Pseudotime trajectory root defined by the acinar (*2*) cluster of 8-week-old Cre mice. Expression of acinar (*Cpa1*) and PanIN (*Gkn2*) markers. (**G**) Chord diagram visualising the upregulated signalling in ductal cells of 8-week-old and 18-week-old KC-Cpa1 mice compared with 8-week-old and 15-week-old KC mice. (**H**) Violin plots depicting iDucts (inflammatory ducts) marker expression in ductal cells of 8-week-old and 18-week-old KC-Cpa1 mice compared with 8-week-old and 15-week-old KC mice. AP-1, activating protein 1; DC, dendritic cell; RBC, red blood cell.

We then turned to the epithelial compartment of KC-Cpa1 mice in single-cell data, which profoundly reflected the histology of KC-Cpa1 pancreata, with negligible acinar cells and advanced metaplasia (figure 4A,B). In comparison to KC mice at similar ages, KC-Cpa1 pancreata displayed an increase in both ADM and PanIN proportions.

As we found *Cpa1^N256K^* to enhance *Kras^G12D^*-induced acinar dedifferentiation (online supplemental figures 1-4) and carcinogenesis (online supplemental table 2) *in vivo*, we sought to validate these findings *ex vivo*. To this end, we isolated acini of KC mice and KC-Cpa1 mice at the age of 8 weeks and embedded them in collagen, followed by an incubation with TGF-α (transforming growth factor alpha) and caerulein (online supplemental materials). Induction of ADM was monitored by live-cell imaging over a time period of 4 days (figure 4C), and the time-dependent increase of ADM area was quantified (online supplemental figure 8). KC-Cpa1 acini displayed enhanced ADM formation compared with KC, indicating an acinar-intrinsic acceleration of carcinogenesis through *Cpa1^N256K^*.

To characterise the acinar-intrinsic interplay of *Cpa1^N256K^* and *Kras^G12D^,* we next juxtaposed KC-Cpa1 ADM with both KC and Cpa1 ADM in single-cell data. Differential gene expression analysis revealed ADM in KC-Cpa1 to differ from Cpa1 in key acinar, ductal and ADM markers, more closely resembling the late ADM stage observed in KC (online supplemental table 7). Moreover, ADM in KC-Cpa1 differed from KC in key markers characterised in Cpa1, underscoring the molecular effects of *Cpa1^N256K^* on ADM dedifferentiation. We then performed a receiver operating characteristic (ROC) analysis to identify genes whose expression would discriminate KC-Cpa1 from the union of KC and Cpa1, revealing additive and inverse additive effects (online supplemental table 8).

In the ROC analysis, we found numerous members of the activating protein 1 (AP-1) transcription factor (TF) family (eg, *Junb*, *Fosb*, *Fos*) to have the most discriminating power in KC-Cpa1 compared with the other genotypes. Thus, AP-1 TFs in KC-Cpa1 saw a stark increase in expression compared with KC and Cpa1 (figure 4D), suggesting a synergistic effect of both genotypes. AP-1 TFs are known downstream targets of Ras^51 52^ and have previously been suggested to cooperate with *Kras^G12D^*-induced ADM.^53^,^55^ In GSEA of RNA-seq samples, *Kras*-signalling was enriched in Cpa1 compared with Cre alone (figure 4E). We thus hypothesised that AP1-dependent *Kras* signalling in Cpa1 would cooperate with mutant *Kras* in early carcinogenesis to amplify *Kras*-induced dedifferentiation. Fittingly, we found *Kras*-signalling to be upregulated in KC-Cpa1 pancreata compared with Cpa1.

To provide a comprehensive resource of inflammation-driven carcinogenesis, we integrated single-cell data of KC-Cpa1 mice at ages 8 weeks, 18 weeks and 52 weeks. We then inferred a pseudotime trajectory through the metaplastic clusters, with the root defined as the acinar cells of 8-week-old Cre mice. We found the inferred trajectory to faithfully capture the progression of acinar cells to ADM and ultimately PanIN (figure 4F). To determine gradual changes of gene expression in pancreatic carcinogenesis, we then performed a Moran’s I test statistic along the inferred trajectory, capturing key markers of pancreatic carcinogenesis described in literature, including acinar, ADM and PanIN markers (online supplemental table 9).

We then sought to examine the contribution of the iDucts identified in Cpa1 mice to the observed phenotype of accelerated ADM and PanIN remodelling in KC-Cpa1. We assessed signalling interactions in the ductal cells of KC-Cpa1 compared with KC mice and found upregulated interaction of proinflammatory genes such as *C3* (complement C3) and *Mif* (macrophage migration inhibitory factor) with granulocytes, macrophages and fibroblasts (figure 4G). Finally, we confirmed high expression levels of iDucts marker genes (eg, *Il1rn, C3, Mmp7*) observed in Cpa1 versus Cre in KC-Cpa1 versus KC mice, reinforcing an inflammatory phenotype of the ductal compartment in early pancreatic carcinogenesis (figure 4H).

### Extensive cell-cell communication networks drive pancreatic inflammation and carcinogenesis

**Figure 5:**
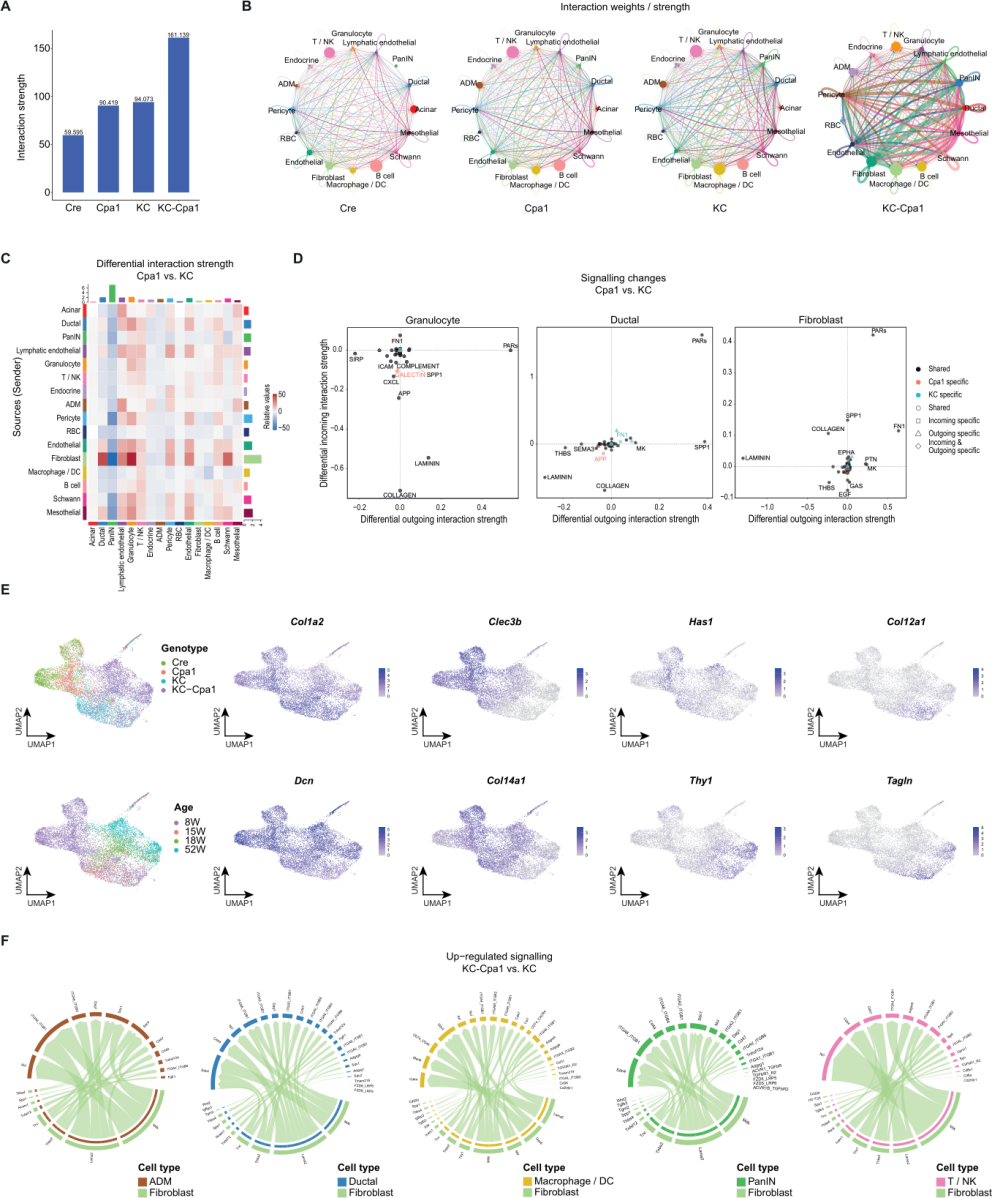
Cell-cell communication networks underlying pancreatic inflammation and early carcinogenesis. (**A**) Interaction strength of inferred cell-cell communication networks in 8-week-old Cre (*Ptf1a^+/Cre^*), Cpa1 (*Cpa1^N256K/N256K^*), KC (*Ptf1a^+/Cre^Kras^LSLG12D/+^*) and KC-Cpa1 (*Ptf1a^+/Cre^Kras^LSLG12D/+^Cpa1^N256K/N256K^*) mice. (**B**) Circle plots visualising the aggregated cell-cell communication network in 8-week-old Cre, Cpa1, KC and KC-Cpa1 mice. (**C**) Heatmap depicting differential interaction strength among cell populations in 8-week-old Cpa1 and KC mice. (**D**) Scatter plots visualising signalling changes between granulocytes, ductal cells and fibroblasts in 8-week-old Cpa1 and KC mice. (**E**) UMAP (uniform manifold approximation and projection) plots depicting expression of fibroblast (*Col1a2*, *Dcn*), iCAF (*Clec3b*, *Col14a1*, *Has1*) and myCAF (*Thy1*, *Col12a1*, *Tagln*) markers across genotype and age. (**F**) Chord diagrams visualising the upregulated signalling in 8-week-old and 18-week-old KC-Cpa1 mice compared with 8-week-old and 15-week-old KC mice. ADM, acinar-to-ductal metaplasia; DC, dendritic cell; iCAF, inflammatory cancer-associated fibroblast; myCAF, myofibroblastic cancer-associated fibroblast; PanIN, pancreatic intraepithelial neoplasia; PARs, protease-activated receptors; RBC, red blood cell.

After studying plasticity in the exocrine compartment, we turned to cell-cell communication networks across ages and genotypes. We first compared the overall strength of cell-cell interactions in Cre, Cpa1, KC and KC-Cpa1 (figure 5A). Here, we found an increase in interaction strength between Cpa1 and KC compared with Cre, and an even further increase in KC-Cpa1. Aggregated cell-cell interactions between the annotated cell types revealed this increased signalling to be carried by both epithelial and stromal cell types, with fibroblasts showing the most significant increase in signalling (figure 5B).

To juxtapose the cell-cell communication networks underlying pancreatic inflammation and carcinogenesis, we then compared the differential interaction strength of cell types in Cpa1 and KC (figure 5C). Here, we found the most significant changes in signalling to occur in granulocytes, ductal cells and fibroblasts. In line with their central role in fibrotic remodelling, fibroblast signalling in Cpa1 was downregulated, in contrast to the upregulated signalling of granulocytes and ductal cells. Across cell types, we found protease-activated receptors (PARs) to strongly contribute to signalling in Cpa1 (figure 5D). PAR signalling is implicated in numerous processes such as inflammation, tissue remodelling and fibrosis.^56 57^ In ductal cells, we again found *Spp1* (figure 2H) to strongly contribute to *Cpa1^N256K^*-induced signalling, underscoring the significance of the iDucts phenotype. Correspondingly, we found significant ingoing *Spp1* signalling in fibroblasts, suggesting a role for *Spp1* in the interaction between ductal cells and fibroblasts.

In pancreatic cancer, cancer-associated fibroblasts (CAFs), tumour cells and immune cells in the tumour microenvironment are characterised by close interaction, with CAFs secreting growth factors, remodelling the ECM and regulating immunity.^58^ Particularly, tumour-suppressing iCAFs and tumour-promoting myofibroblastic CAFs (myCAFs) have been studied in pancreatic cancer.^59 60^ As we studied CAF signatures in the fibroblast cluster, we identified extensive differences in marker expression across genotypes (figure 5E). In Cre and Cpa1 mice, we found fibroblasts to display iCAF marker expression, while KC was characterised by both iCAFs and myCAFs. Strikingly, we found fibroblasts in KC-Cpa1 to predominantly display myCAF marker expression, suggesting a link between inflammation, early carcinogenesis and CAF subtypes. To study the impact of *Cpa1^N256K^* on fibroblast signalling in carcinogenesis, we compared fibroblast signalling in KC-Cpa1 to KC (figure 5F, online supplemental table 10). Notably, we observed increased signalling in KC-Cpa1 fibroblasts, particularly in genes associated with ECM remodelling, such as *Lama2*^61^ (laminin subunit alpha 2), further highlighting the role of fibroblast-driven remodelling in early pancreatic carcinogenesis.

### Discussion

CP is a major risk factor for pancreatic cancer development. Here, we demonstrated that the CP mutation *Cpa1^N256K^* accelerates early carcinogenesis in the KC mouse model, with an increase of ADM and PanIN formation, and profound tissue remodelling. We found *Cpa1^N256K^* to induce extensive plasticity in both the acinar and ductal compartments, including an early ADM state in acinar cells characterised by an upregulation of ER stress markers and iDucts, inflammatory ductal cells. We demonstrated how extensive cell-cell interaction networks contribute to inflammation and carcinogenesis, and described key properties of the inflammatory KC-Cpa1 phenotype.

To uncover how *Cpa1^N256K^*-induced plasticity accelerates *Kras^G12D^* remodelling, we compared the expression levels of top differentially expressed genes in the respective ADM compartments. In Cpa1 ADM, we found the expression of *Klk1* (kallikrein 1) to be most significantly upregulated, a member of Kallikrein-related peptidases implicated in remodelling of the tumour microenvironment.^62^ We also found upregulation of the pancreatitis-inducible small chromatin protein *Nupr1* (nuclear protein 1) in Cpa1 ADM. *Nupr1* is a basic helix–loop–helix molecule strongly induced in acute pancreatitis^63 64^ and overexpressed in various human cancers, including PDAC. Notably, *Kras^G12D^;Nupr1^-/-^* mice were found to be protected from the development of PanIN lesions^65^ by modulating *Kras^G12D^*-induced senescence.^66^ We also found a stark increase of *Nupr1* in KC-Cpa1 ADM compared with KC, suggesting *Kras^G12D^*-induced formation of PanIN lesions to be enhanced by an inflammation-induced stress response dependent on *Nupr1*.

We found AP-1 TFs (eg, *Jun*, *Junb*, *Fos*) to hold significant discriminative power in an ROC analysis of KC-Cpa1 ADM as compared with both Cpa1 and KC, revealing a synergistic effect of the inflammatory and oncogenic transcriptional programmes. In *Nr5a2^+/−^*mice, *Jun* deletion rescued the *Nr5a2^+/−^*-induced proinflammatory phenotype, underscoring its significance in pancreatic inflammation.^67^ AP-1 TFs are known downstream targets of Ras,^51 52^ and previous studies have suggested members of the AP-1 complex to cooperate with mutant Kras to drive ADM and carcinogenesis in the pancreas.^53 55^ *Kras^G12D^* and tissue damage promoted a chromatin state in acinar cells characterised by a switch to open-chromatin regions enriched for AP-1 motifs, which was found to be selected throughout the malignant transformation.^53^ Similarly, *Kras^G12D^* has been shown to stabilise an enhancer-network including AP-1 factors such as *Junb* and *Fosl1*, locking acinar cells in a pancreatic progenitor state susceptible to initiation of tumorigenesis, while depletion of *Junb* and *Fosl1* blocked pancreatic cancer progression. Notably, this enhancer network was also found to distinguish ductal subpopulations in human PDAC tumours.^55^ *JUNB* (classical) and *cJUN* (basal-like) driven regulation of PDAC subtypes further suggested the *cJUN/TNF-α* high subtype as a target of highly inflamed and aggressive PDAC states.^68 69^ *Fosl1* has recently been found to be the most active TF in *Kras^G12D^* mice undergoing acute pancreatitis, while acinar-specific *Fosl1* knockout in *Kras^G12D^* mice led to a delayed onset of ADM and neoplastic transformation.^55^ Thus, *Cpa1^N256K^* may sensitise acinar cells to mutant *Kras* by inducing an early ADM transcriptional programme characterised by a pronounced upregulation of AP-1 and *Kras* signalling.

In both Cpa1 and KC-Cpa1, scRNA-seq revealed a subtype of inflammatory ductal cells (iDucts). We found this population to be different from previously described ductal subtypes in acute pancreatitis and carcinogenesis,^46^ suggesting disease-specific properties of ductal cells. Among the top upregulated genes in iDucts were *Cxcl5* and *C3*, both linked to neutrophil infiltration in PDAC^70 71^ and acute pancreatitis.^72^ In line with these findings, we found upregulated signalling of iDucts to granulocytes, macrophages and fibroblasts, particularly for *Spp1*.^46 47^ The iDucts marker Basp1 has previously been identified as a potential biomarker in patients with pancreatic cancer.^73^ Although it has been experimentally demonstrated that ductal cells can give rise to PDAC,^74^ our understanding of ductal plasticity and metaplasia remains limited. Studies on ductal dedifferentiation in early carcinogenesis and the relation of ductal subtypes to PDAC will deepen our understanding of the earliest stages of pancreatic cancer development.

Across genotypes, we found inflammation and carcinogenesis to be accompanied by extensive cell-cell communication network activity. KC-Cpa1 displayed particularly strong interaction, in line with the drastic remodelling witnessed in histology. As we compared cell signalling in Cpa1 and KC, the interaction of fibroblasts, granulocytes and ductal cells was particularly upregulated, underscoring the significance of the iDucts phenotype in inflammation. Strikingly, marker expression in fibroblasts shifted from a predominance of iCAF markers in Cpa1 to myCAF markers in KC-Cpa1. This is particularly interesting considering the essential role of CAFs in the tumour microenvironment, their implications on therapy resistance and the correlation of CAF subtypes and prognosis.^59 60^ We also found this effect in KC and KC-Cpa1, suggesting a link between inflammation and CAF subtypes in metaplasia. Among the genes upregulated in KC-Cpa1 fibroblast signalling, we found *Lama2* (laminin subunit alpha 2) to show a particularly strong interaction with other cell types. As a major component of the basement membrane involved in attachment, migration and ECM organisation, *Lama2* has recently been shown to suppress cancer cell migration, proliferation and invasion.^61 75^

In the recently reported mouse model *Ctrb1^Δexon6^* carrying a human pancreatitis-associated risk variant *CTRB2^Δexon6^*, accumulation of misfolded Ctrb1 resulted in an upregulation of ER stress and unfolded protein response (UPR) pathways accompanied by a downregulation of the acinar differentiation programme.^76^ Interestingly, ER stress inhibition by TUDCA (tauroursodeoxycholic acid) and Sulindac as candidate therapeutic strategies revealed rather modest effects in *Ctrb1^Δexon6^*, warranting further studies on therapeutic intervention in *Ctrb1^Δexon6^* and *Cpa1^N256K^*. It will be intriguing to unveil whether *Ctrb1^Δexon6^* and other recently developed mouse models of hereditary pancreatitis^15^,^24^ show similar sensitisation to *Kras^G12D^* transformation, thus revealing conserved mechanisms in the interplay of hereditary CP and carcinogenesis. As these models can capture the constant, intrinsic stress characterising the pathophysiology of hereditary CP, they will complement the well-established models of repeated caerulein injections. This is particularly relevant, considering that significant proportions of patients develop CP without prior episodes of acute pancreatitis,^77 78^ and caerulein models are based on the supraphysiological stimulation of acinar cells.

In summary, we present the first model capturing the interplay of hereditary CP and pancreatic carcinogenesis. It robustly recapitulates early stages of pancreatic cancer development and offers insight into the complex inflammatory networks underlying carcinogenesis. Thus, this model holds potential for the discovery of relevant biomarkers and therapeutic targets.

## Supplementary material

10.1136/gutjnl-2025-335947 online supplemental figure 1

10.1136/gutjnl-2025-335947 online supplemental figure 2

10.1136/gutjnl-2025-335947 online supplemental figure 3

10.1136/gutjnl-2025-335947 online supplemental figure 4

10.1136/gutjnl-2025-335947 online supplemental figure 5

10.1136/gutjnl-2025-335947 online supplemental figure 6

10.1136/gutjnl-2025-335947 online supplemental figure 7

10.1136/gutjnl-2025-335947 online supplemental figure 8

10.1136/gutjnl-2025-335947 online supplemental table 1

10.1136/gutjnl-2025-335947 online supplemental table 2

10.1136/gutjnl-2025-335947 online supplemental table 3

10.1136/gutjnl-2025-335947 online supplemental table 4

10.1136/gutjnl-2025-335947 online supplemental table 5

10.1136/gutjnl-2025-335947 online supplemental table 6

10.1136/gutjnl-2025-335947 online supplemental table 7

10.1136/gutjnl-2025-335947 online supplemental table 8

10.1136/gutjnl-2025-335947 online supplemental table 9

10.1136/gutjnl-2025-335947 online supplemental table 10

10.1136/gutjnl-2025-335947 online supplemental file 1

10.1136/gutjnl-2025-335947 online supplemental file 2

## Data Availability

Data are available in a public, open access repository. All data relevant to the study are included in the article or uploaded as supplementary information.
