# Supplementary material for: Hereditary chronic pancreatitis induced plasticity cooperates with mutant Kras in early pancreatic carcinogenesis
Source: Gut. 2025 Dec 19;75(5):e335947. doi: 10.1136/gutjnl-2025-335947 (PMC13151493; doi:10.1136/gutjnl-2025-335947)
Supplement: online supplemental figure 3 [file gutjnl-75-5-s003.pdf]

Online supplemental figure 3

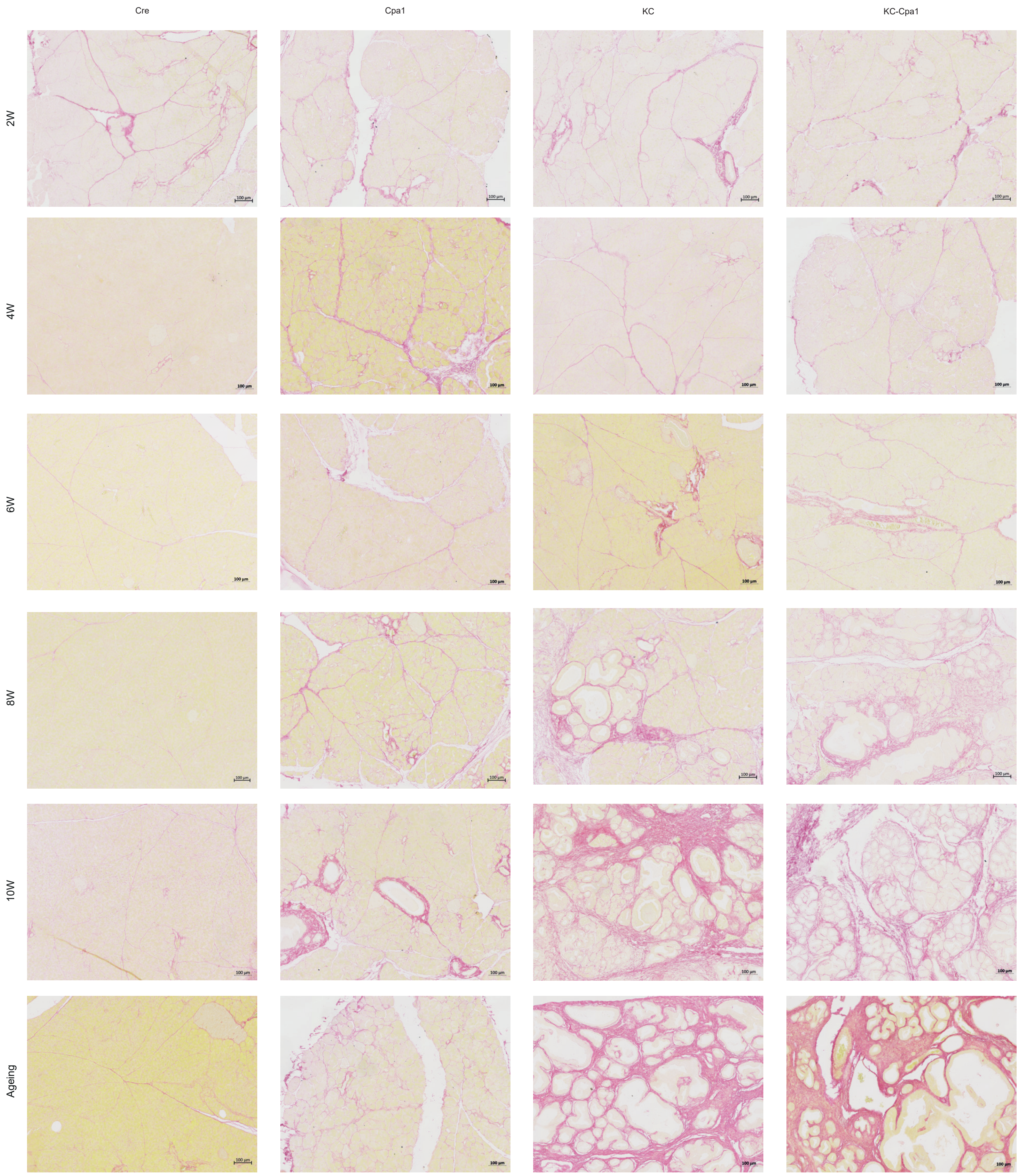

Online supplemental figure 3 Sirius red staining. Representative images of Sirius red stained pancreas sections from Cre (*Ptf1a*$^{+/Cre}$), Cpa1 (*Cpa1*$^{N256K/N256K}$), KC (*Ptf1a*$^{+/Cre}$*Kras*$^{LSLG12D/+}$) and KC-Cpa1 (*Ptf1a*$^{+/Cre}$*Kras*$^{LSLG12D/+}$ *Cpa1*$^{N256K/N256K}$) mice at 2, 4, 6, 8, 10 weeks and ageing time points. Images of 8 weeks correspond to main figure 1B.
