# Supplementary material for: Hereditary chronic pancreatitis induced plasticity cooperates with mutant Kras in early pancreatic carcinogenesis
Source: Gut. 2025 Dec 19;75(5):e335947. doi: 10.1136/gutjnl-2025-335947 (PMC13151493; doi:10.1136/gutjnl-2025-335947)
Supplement: online supplemental figure 4 [file gutjnl-75-5-s004.pdf]

# Online supplemental figure 4

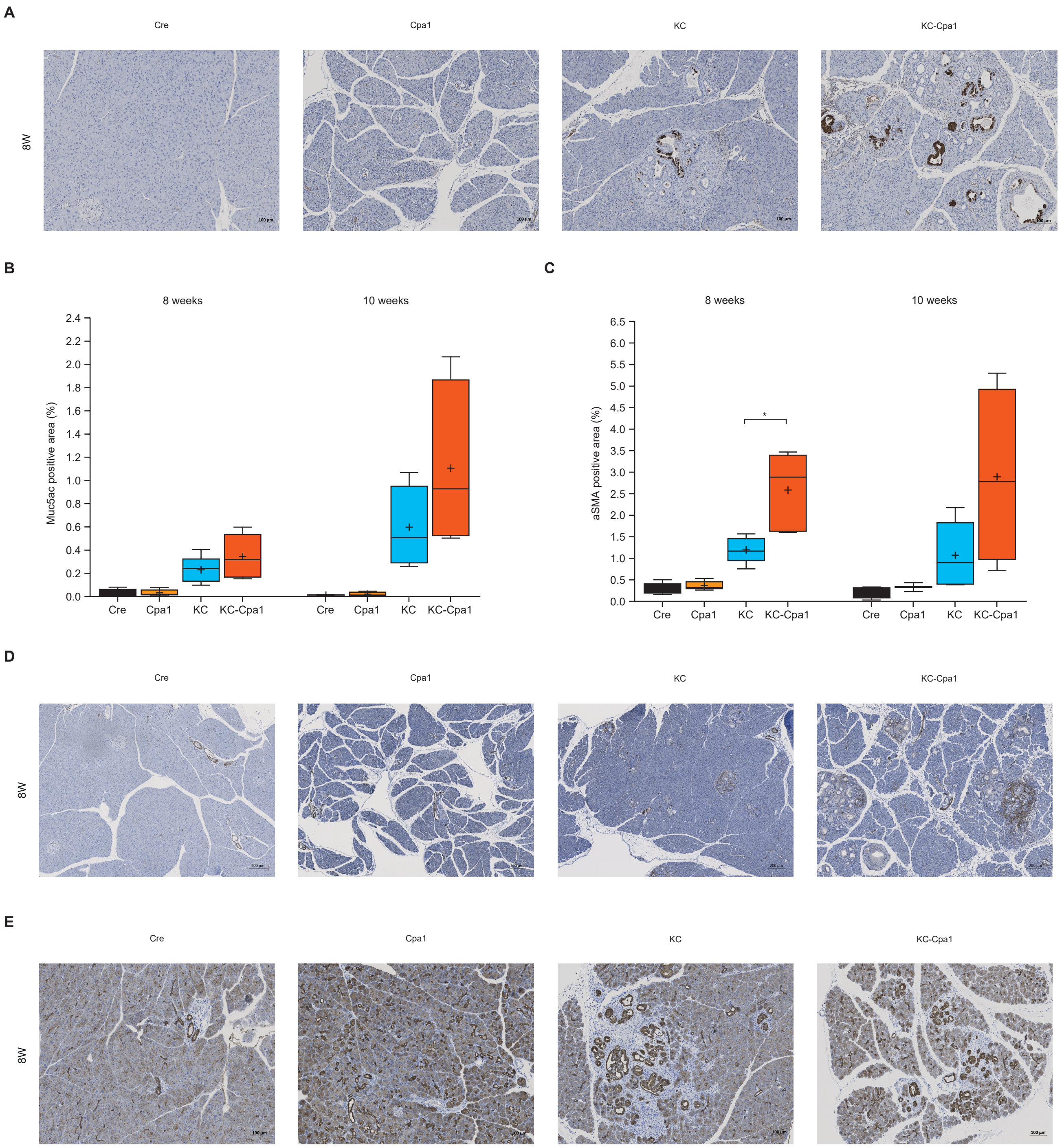

**Online supplemental figure 4** Immunohistochemistry ADM and PanIN. Representative images of (A) Muc5ac, (D) α-SMA and (E) Krt19 staining in the pancreata of 8-week-old Cre (*Ptf1a*$^{+/Cre}$), Cpa1 (*Cpa1*$^{N256K/N256K}$), KC (*Ptf1a*$^{+/Cre}$*Kras*$^{LSLG12D/+}$) and KC-Cpa1 (*Ptf1a*$^{+/Cre}$*Kras*$^{LSLG12D/+}$*Cpa1*$^{N256K/N256K}$) mice. Quantification of (B) Muc5ac (8W: n=5, 10W: Cre, Cpa1, KC: n=5, KC-Cpa1: n=4) and (C) α-SMA (8W: n=5, 10W: Cre: n=4, Cpa1: n=2, KC: n=5, KC-Cpa1: n=4) staining in Cre, Cpa1, KC and KC-Cpa1 mice through a pixel classifier of stained areas. Statistical comparison using t-test (* $p<0.05$).
