# Supplementary material for: Hereditary chronic pancreatitis induced plasticity cooperates with mutant Kras in early pancreatic carcinogenesis
Source: Gut. 2025 Dec 19;75(5):e335947. doi: 10.1136/gutjnl-2025-335947 (PMC13151493; doi:10.1136/gutjnl-2025-335947)
Supplement: online supplemental figure 5 [file gutjnl-75-5-s005.pdf]

# Online supplemental figure 5

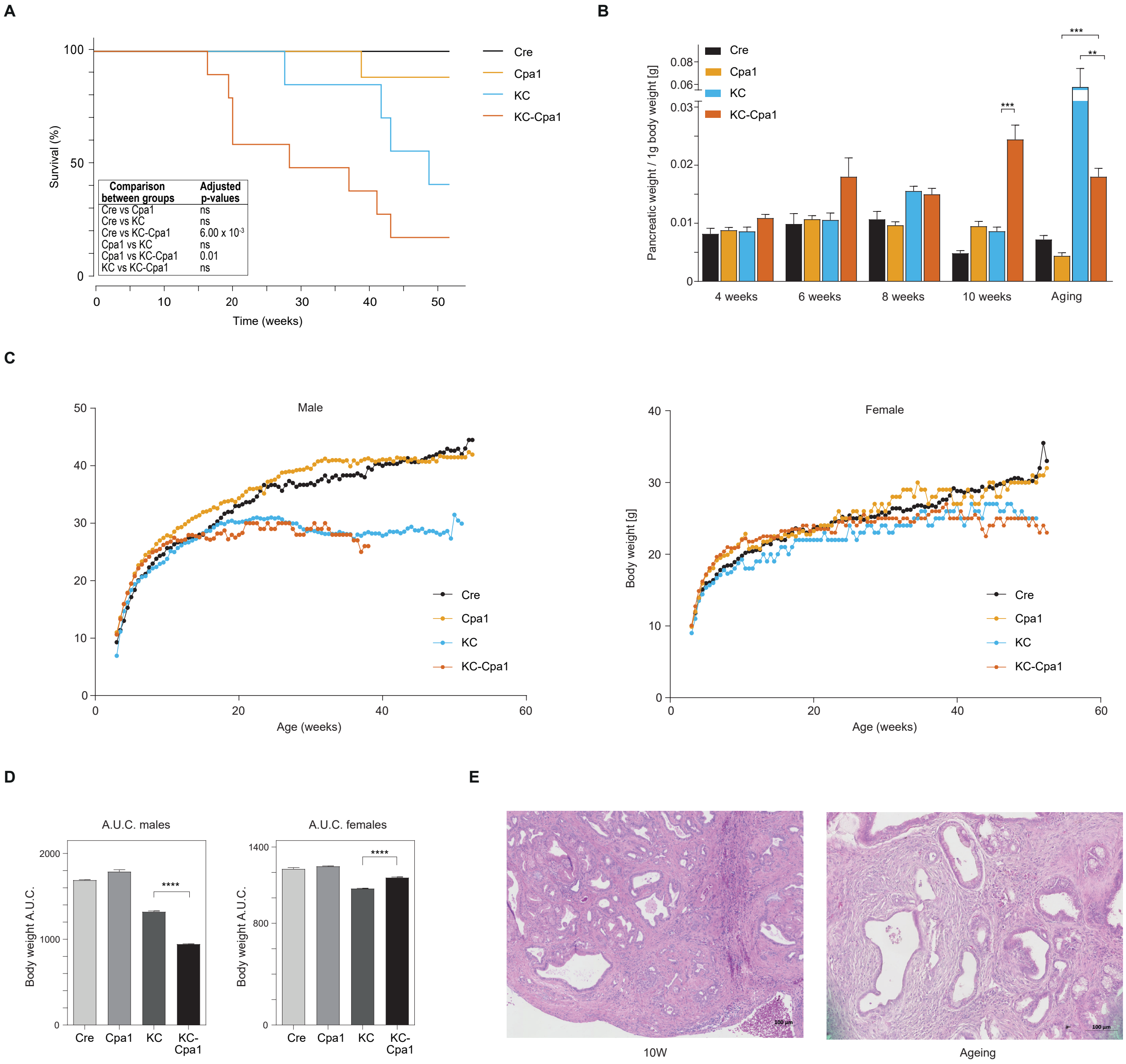

**Online supplemental figure 5** Phenotypic characterisation of Cre, Cpa1, KC and KC-Cpa1 mice. (A) Kaplan-Meier survival curve over 52-week period in Cre (*Ptf1a*$^{+/Cre}$) mice (n=8), Cpa1 (*Cpa1*$^{N256K/N256K}$) mice (n=9), KC (*Ptf1a*$^{+/Cre}$*Kras*$^{LSLG12D/+}$) mice (n=7) and KC-Cpa1 (*Ptf1a*$^{+/Cre}$*Kras*$^{LSLG12D/+}$ *Cpa1*$^{N256K/N256K}$) mice (n=10). Mice were euthanised upon reaching pre-defined humane endpoint criteria - such as changes in appearance, behaviour, body weight, or tumour size. Statistical comparison using log-rank test with Bonferroni adjusted p-values ($p < 0.05$). (B) Bar plots depicting pancreas weight relative to the body weight over time. Statistical comparison using t-test (** $p<0.01$, *** $p<0.001$, n=5 per genotype per time point). (C) Body weight in male and female mice over time. Body weight was measured at defined time points using all available animals per genotype: five mice per group at 2, 4, 6, 8 and 10 weeks and the full ageing cohort (A). (D) AUC (area under the curve) analysis of body weight (C) in male and female mice. Statistical comparison using t-test (**** $p<0.0001$). (E) Representative H&E staining of cancer in KC-Cpa1 pancreas sections.
