# Supplementary material for: Hereditary chronic pancreatitis induced plasticity cooperates with mutant Kras in early pancreatic carcinogenesis
Source: Gut. 2025 Dec 19;75(5):e335947. doi: 10.1136/gutjnl-2025-335947 (PMC13151493; doi:10.1136/gutjnl-2025-335947)
Supplement: online supplemental figure 6 [file gutjnl-75-5-s006.pdf]

Online supplemental figure 6

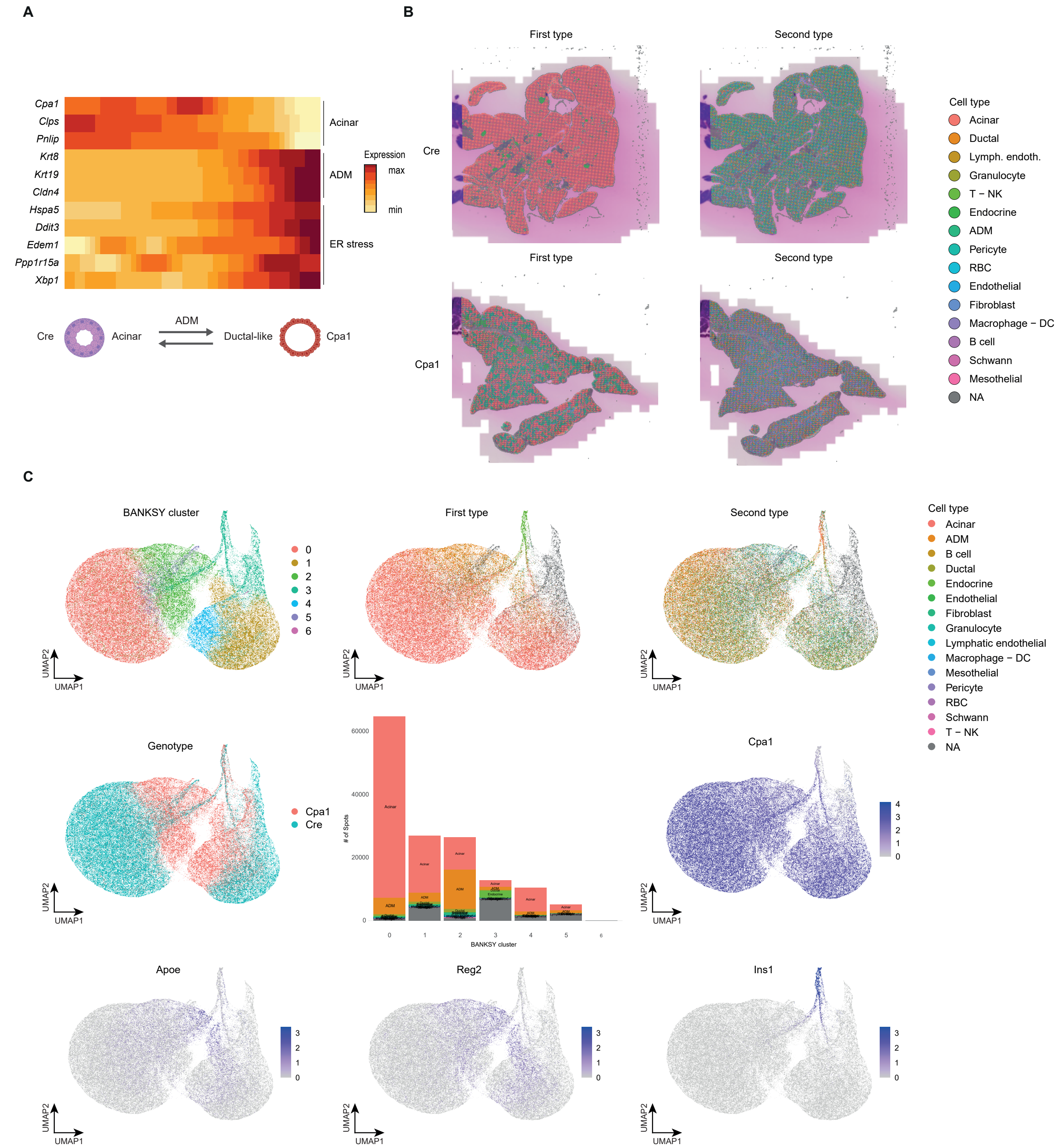

Online supplemental figure 6. Acinar plasticity in Cpa1 mice. (A) Heatmap depicting expression of acinar, ADM and ER stress markers as a function of pseudotime through the acinar compartment of 8-week-old Cre (*Ptf1a*$^{+/Cre}$) and Cpa1 (*Cpa1*$^{N256K/N256K}$) mice. (B) H&E staining and spatial deconvolution of 8-week-old Cpa1 and Cre pancreata, based on single-cell data of Cre and Cpa1 mice at corresponding ages. (C) UMAP plots depicting clusters and marker expression in tissue domains of Cpa1 and Cre samples predicted using BANKSY.
