# Supplementary material for: Hereditary chronic pancreatitis induced plasticity cooperates with mutant Kras in early pancreatic carcinogenesis
Source: Gut. 2025 Dec 19;75(5):e335947. doi: 10.1136/gutjnl-2025-335947 (PMC13151493; doi:10.1136/gutjnl-2025-335947)
Supplement: online supplemental figure 7 [file gutjnl-75-5-s007.pdf]

Online supplemental figure 7

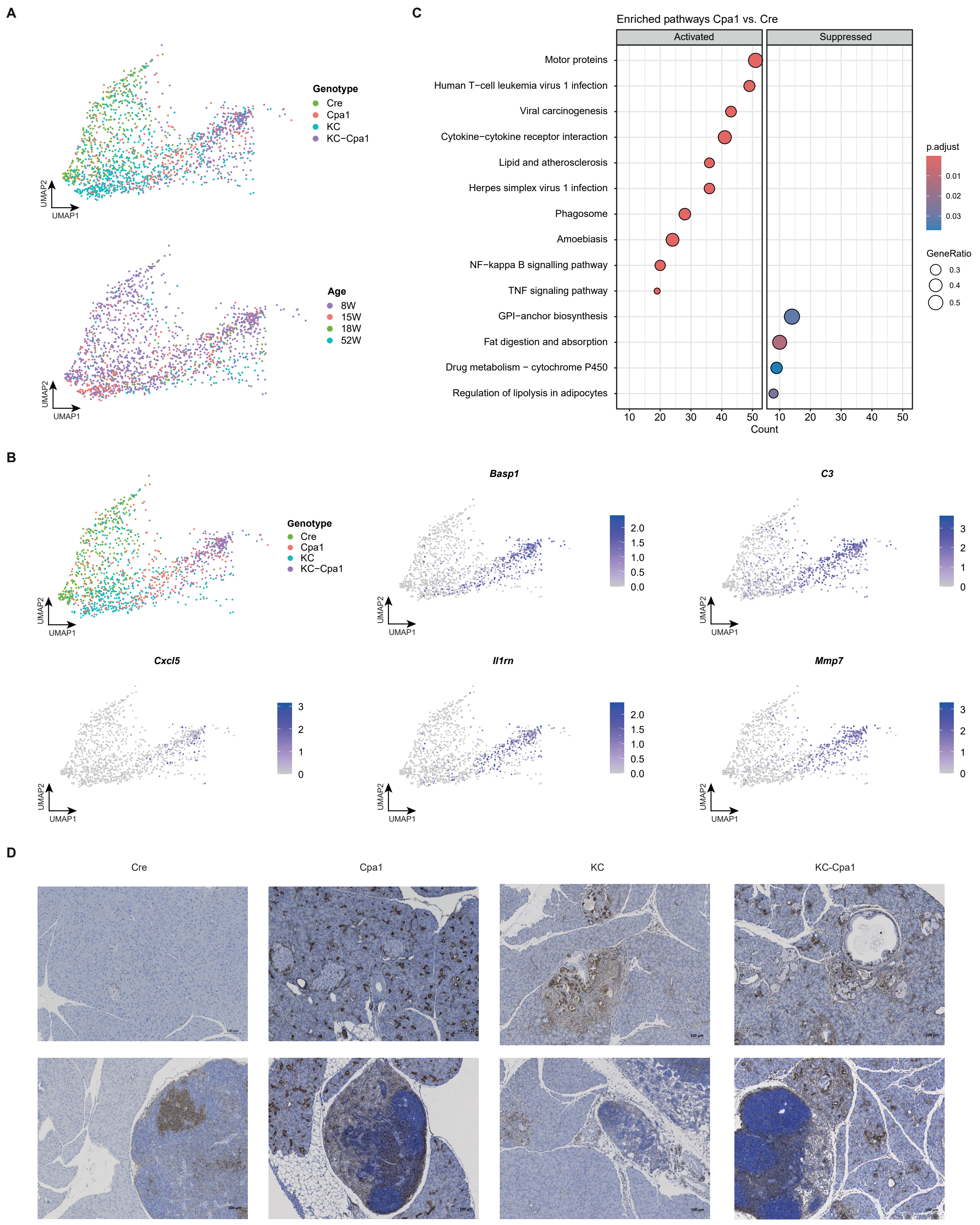

Online supplemental figure 7 Ductal compartment across genotypes and ages. (A) UMAP plots depicting the ductal compartment of Cre (*Ptf1a*$^{+/Cre}$), Cpa1 (*Cpa1*$^{N256K/N256K}$), KC (*Ptf1a*$^{+/Cre}$*Kras*$^{LSLG12D/+}$) and KC-Cpa1 (*Ptf1a*$^{+/Cre}$*Kras*$^{LSLG12D/+}$ *Cpa1*$^{N256K/N256K}$) mice across ages. (B) UMAP plots depicting the ductal compartment of 8-week-old Cre, Cpa1, KC and KC-Cpa1 mice and iDuct (inflammatory duct) marker expression. (C) Gene set enrichment analysis (GSEA) of the ductal compartment in 8-week-old Cpa1 mice compared to Cre. (D) Representative images of Basp1 staining in the epithelial compartment (upper row) and lymphatic tissue (lower row) of 8-week-old Cre, Cpa1, KC and KC-Cpa1 pancreata.
