# Supplementary material for: Hereditary chronic pancreatitis induced plasticity cooperates with mutant Kras in early pancreatic carcinogenesis
Source: Gut. 2025 Dec 19;75(5):e335947. doi: 10.1136/gutjnl-2025-335947 (PMC13151493; doi:10.1136/gutjnl-2025-335947)
Supplement: online supplemental figure 8 [file gutjnl-75-5-s008.pdf]

Online supplemental figure 8

A

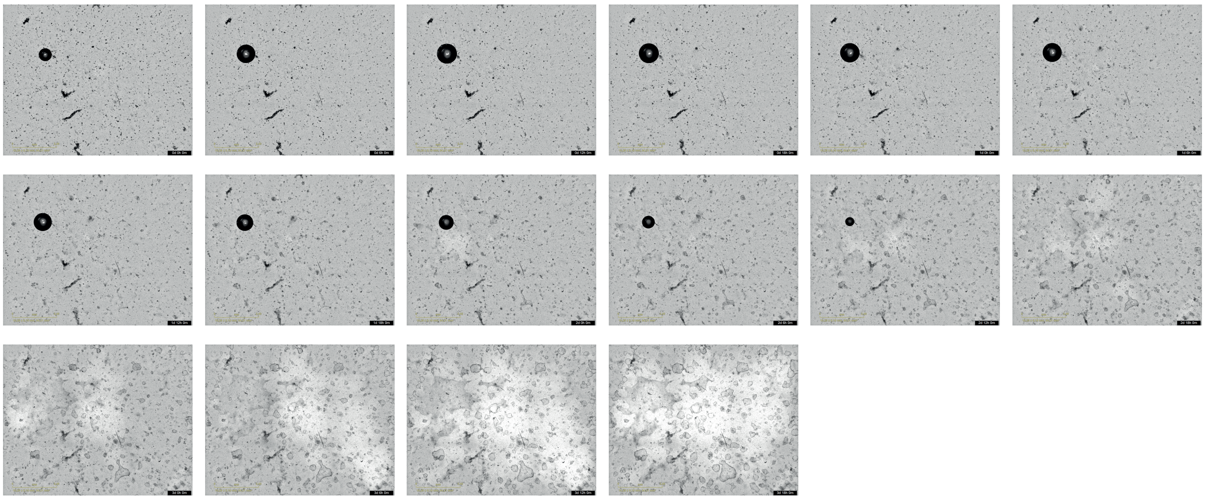

B

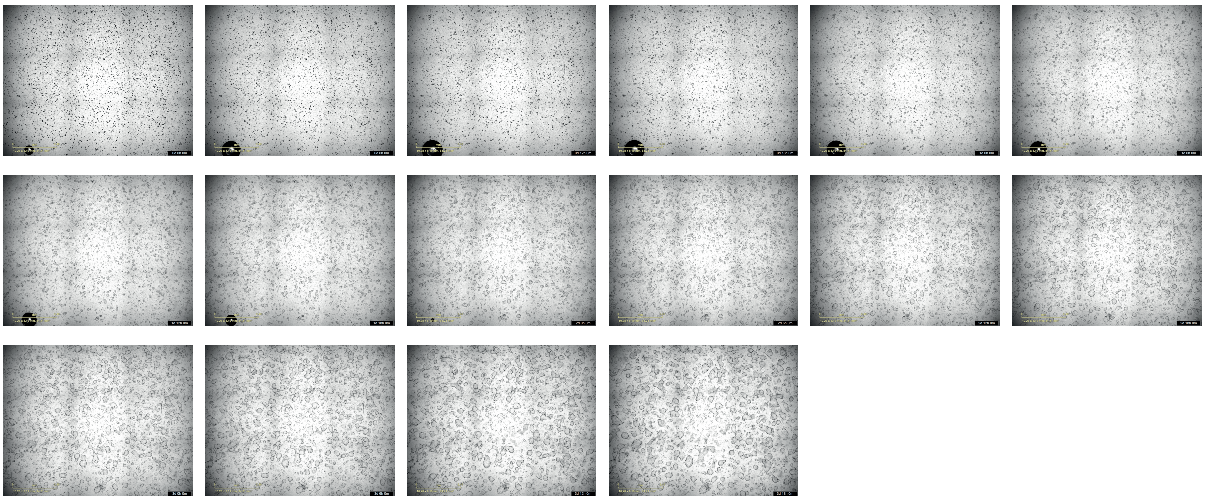

C

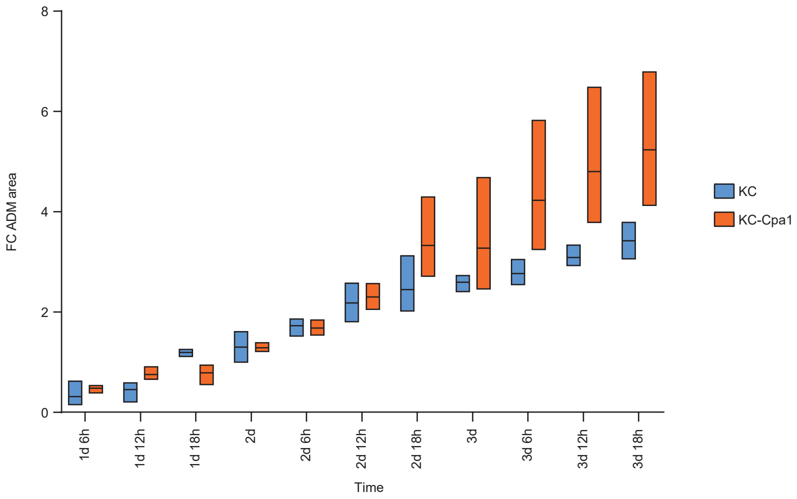

Online supplemental figure 8 Primary acinar transdifferentiation capacity in KC-Cpa1 and KC. (A,B) Representative images of primary acini isolated from KC-Cpa1 (A) and KC (B) mice embedded in collagen, stimulated with TGF-α and caerulein, and monitored by live-cell imaging for four days. (C) Quantification (n=3) of ADM area increase based on a pretrained pixel classifier, normalized to the mean early ADM area. Floating bars represent minimum and maximum values, line at mean. Statistical comparison using t-test with FDR adjusted p-values.
