# Supplementary material for: Hereditary chronic pancreatitis induced plasticity cooperates with mutant Kras in early pancreatic carcinogenesis
Source: Gut. 2025 Dec 19;75(5):e335947. doi: 10.1136/gutjnl-2025-335947 (PMC13151493; doi:10.1136/gutjnl-2025-335947)
Supplement: online supplemental file 1 [file gutjnl-75-5-s019.pdf]

Hereditary Chronic Pancreatitis Induced Plasticity Cooperates with Mutant Kras in Early Pancreatic Carcinogenesis

Key findings

- We introduce a novel humanized mouse model leveraging **chronic pancreatitis** ***Cpa1***^{N256K} and **oncogenic** ***Kras***^{G12D} mutations and by single-cell RNA-sequencing unveil the impact of **chronic inflammation** on **early carcinogenesis.**
- Cpa1N256K mice exhibit exocrine plasticity marked by an **early ADM state** and inflammatory ductal cells (**iDucts**)
- In *Cpa1*^{N256K}*Kras*^{G12D} mice we find that chronic inflammation accelerates development of PanIN lesions and fibrosis.
- We report rewiring of the intercellular communication-networks of **ductal**, **fibroblast** and **granulocyte** cells accompanied with enhanced **myCAF**

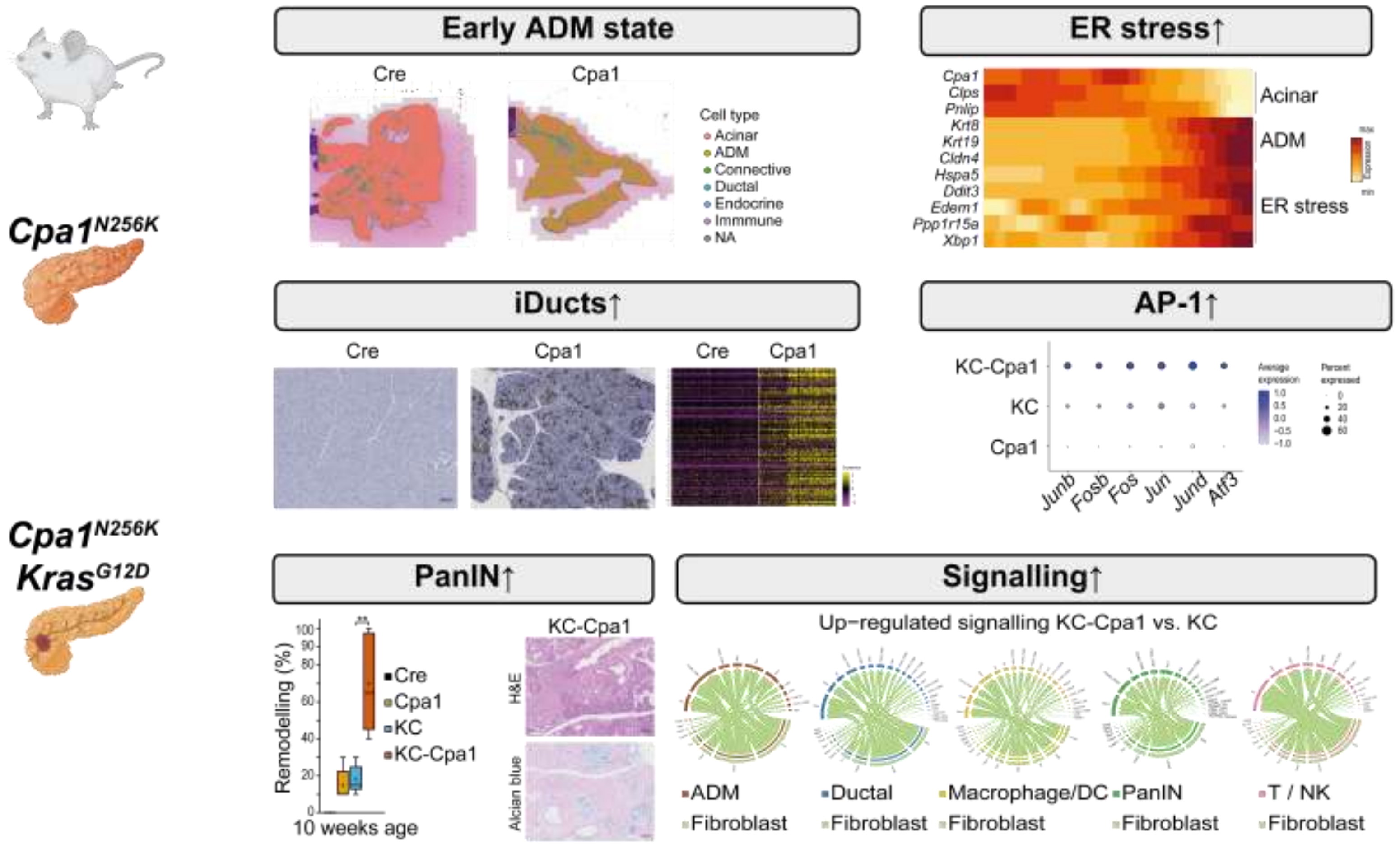

Inamdar, Krannich, Hesselbarth et al. *Gut* 2025. DOI: 10.1136/gutjnl-2025-335947

BMJ bsg gut.bmj.com @Gut_BMJ Gut
