# Supplementary material for: Hereditary chronic pancreatitis induced plasticity cooperates with mutant Kras in early pancreatic carcinogenesis
Source: Gut. 2025 Dec 19;75(5):e335947. doi: 10.1136/gutjnl-2025-335947 (PMC13151493; doi:10.1136/gutjnl-2025-335947)
Supplement: online supplemental file 2 [file gutjnl-75-5-s020.pdf]

SUPPLEMENTAL MATERIALS

Mouse models and breeding strategy

The generation of Cre (*Ptf1a*$^{+/Cre}$), KC (*Ptf1a*$^{+/Cre}$*Kras*$^{LSLG12D/+}$), and Cpa1 (*Cpa1*$^{N256K/N256K}$) mouse strains has been previously described[1–3]. To generate the compound mutant KC-Cpa1 strain, *Kras*$^{LSL.G12D/+}$ or *Ptf1a*$^{+/Cre}$ mice were first crossed each with *Cpa1*$^{N256K/N256K}$ mice, all maintained on a *C57BL/6N* genetic background. In the F1 generation, offspring harbouring either *Kras*$^{LSL.G12D/+}$*Cpa1*$^{N256K/WT}$ or *Ptf1a*$^{+/Cre}$*Cpa1*$^{N256K/WT}$ genotypes were obtained. These animals were subsequently bred with *Cpa1*$^{N256K/N256K}$ homozygous mice. The resulting progeny included *Kras*$^{LSL.G12D/+}$*Cpa1*$^{N256K/N256K}$ or *Ptf1a*$^{+/Cre}$*Cpa1*$^{N256K/N256K}$ genotypes, which were interbred to obtain triple mutant mice *Ptf1a*$^{+/Cre}$*Kras*$^{LSLG12D/+}$ *Cpa1*$^{N256K/N256K}$, referred to as KC-Cpa1. These animals simultaneously express mutant *Kras* and *Cpa1* in a pancreas-specific manner.

Genotyping of the mice

Genomic DNA was isolated from ear punch biopsies using PBND buffer (50 mM KCl, 10 mM Tris-HCl, pH 8.3, 2.5 mM $MgCl_2 \cdot 6H_2O$, 0.45% v/v NP-40, 0.45% v/v Tween-20) supplemented with proteinase K (200 µg/mL). Genotyping was performed by PCR using the following primer sets: Ptf1a-Cre: Forward: 5′-AACATGCTTCATCGTCGG-3′, Reverse: 5′-TTGCCCCTGTTTCACTATCCAG-3′: Expected product: 700 bp; Kras$^{LSL.G12D}$**:** Forward: 5′-CTAGCCACCATGGCTTGAGT-3′, Reverse: 5′-TCCGAATTCAGTGACTACAGATG-3′: Expected product: 330 bp and Cpa1^{N256K}**:** Forward: 5′-TgAgCCCCACAAgTggAg-3′, Reverse: 5′-gCCCAAgTCCCTgAgTgT-3′: Expected product: 700 bp. PCR was performed in 29 cycles using GoTaq® G2 DNA Polymerase (Promega, M7845) with either 5X Green GoTaq® buffer (for *Ptf1a-Cre* and *Kras*) or 5X Colorless GoTaq® buffer (for *Cpa1*), dNTP Mix (10 mM, Promega, U1511), and primers synthesised by biomers.net. Reactions were run on an Eppendorf Mastercycler® under the following cycling conditions: initial denaturation at 95 °C for 2 minutes, followed by 29 cycles of 95 °C for 30 seconds, 60 °C for 30 seconds, and 72 °C for 1 minute, with a final extension at 72 °C for 2 minutes. PCR products were visualised by gel electrophoresis. To distinguish between wild-type and mutant *Cpa1* alleles, PCR products were subjected to melting curve analysis using a fluorescently labeled hybridisation probe on a LightCycler® system (Roche). The probe sequence was designed to specifically bind the mutant allele: 5′-ggCAgCAXITCCCACTTCCTgTTg--PH-

3′ where "X" represents the site of the mutation. The mutant allele exhibited a distinct melting temperature compared to the wild-type sequence, allowing for precise discrimination of genotypes.

Phenotypic characterisation

All experimental procedures were conducted using both male and female mice. For each time point, genotype, and cohort, at least five animals were included in the analysis. To ensure consistency and minimise genetic variability, all strains were maintained on a pure C57BL/6N background. Due to the specific breeding strategies required for each genotype, Cre and KC mice were derived from one set of breeding pairs, while Cpa1 and KC-Cpa1 mice originated from a separate breeding scheme. Mice were sacrificed at defined time points (2, 4, 6, 8, and 10 weeks of age) via isoflurane overdose (Vetflurane, Virbac), followed by cervical dislocation to ensure euthanasia. A second independent 10-week-old cohort was generated. An additional ageing cohort was monitored longitudinally and sacrificed at 52 weeks of age, or earlier if humane endpoint criteria were met as determined by a standardised health scoring sheet. Body weight was measured biweekly and showed more pronounced weight loss or stagnation in KC and KC-Cpa1 male mice compared to females (supplemental figure 5). This difference may be explained by several factors including (i) anti-inflammatory and antiproliferative effects of estrogens in females, which limit pancreatic lesion development in KC mice and reduce related metabolic stress[4], (ii) greater susceptibility of males to KC-associated pathology, including accelerated ADM and PanIN formation when combined with inflammatory stimuli[5,6], (iii) higher tumour burden in males even under standard chow conditions[7], and (iv) both stronger inflammatory responses and potentially reduced food intake due to dominance-related social stress in group housing[8]. Immediately *post-mortem*, the pancreas, spleen, liver, lung, and a segment of intestine (proximal to duodenum) were harvested. Special care was taken to excise the pancreas rapidly to preserve tissue integrity. Pancreatic tissue was weighed, and the organ-to-body weight ratio was calculated for each animal. When preparing organs, we did not find any metastases in our cohorts by macroscopical assessment of organs. All collected organs were snap-frozen in liquid nitrogen and stored at −80 °C for subsequent analyses.

Histology

Following harvesting, the pancreas was fixed in 4% paraformaldehyde (PFA; ROTI®Histofix; Carl Roth GmbH + Co. KG, P087.1) for 24 hours at room temperature and subsequently transferred to 70% ethanol for storage until further processing. Dehydration was performed using an ascending ethanol

series followed by xylene and paraffin infiltration, using the Epredia™ STP 120 Spin Tissue Processor (Epredia). Tissues were embedded in paraffin with the Microm EC 350 Embedding Centre (Thermo Fisher Scientific). Formalin-fixed paraffin-embedded (FFPE) tissues were sectioned using the Epredia™ HM 340E rotary microtome (Epredia) at 7 µm thickness for all cohorts except for the ageing cohort where 1 µm was applied. One section was mounted per slide. Histological staining was performed on specific serial sections. For all cohorts, H&E staining (Morphisto, 10231.00500; Sigma-Aldrich, HT110116-500ML) was applied to the first, tenth, and twentieth sections, while Alcian Blue (Sigma-Aldrich, TMS-010-C) and Sirius Red (Morphisto, 13422.00500) staining were performed on the second and third sections, respectively. In the ageing cohort, the first, fifth, and tenth sections were stained with H&E, with Alcian Blue and Sirius Red applied to the second and third sections, respectively. For all staining procedures, slides were dewaxed and rehydrated prior to staining. H&E staining was performed manually by dewaxing slides in ROTICLEAR® (2 × 10 minutes) (Carl Roth GmbH + Co. KG, A538.1), followed by rehydration in a graded ethanol series (100%, 96%, 80%, 70%; 5 minutes each) and rinsing in distilled water for 3 minutes. Hematoxylin was applied for 6 minutes, followed by blueing under running tap water (5 minutes). After a brief dip in 96% ethanol (30 seconds), eosin was applied for 1 minute. Slides were rinsed in distilled water, dehydrated in ascending ethanol concentrations (70% for 2 minutes; 80%, 96%, 100% for 1 minute each), cleared in xylene (10 dips and a final rinse), and mounted with ROTI®Mount (Carl Roth GmbH + Co. KG, HP68.1) and a coverslip.

In contrast, H&E staining for the ageing cohort was carried out using the Leica ST5010 Autostainer XL and the ST Infinity 2.0 H&E Staining System (Leica Biosystems) according to the manufacturer's protocol. Slides were subsequently mounted and cover slipped. For Alcian Blue and Sirius Red stainings, slides were dewaxed by incubation at 60°C for 30 minutes, immersed in xylene (3 × 3 minutes), rehydrated through ethanol (100%, 80%, 50%; 3 minutes each), and rinsed in distilled water (2 minutes). Alcian Blue staining included an additional step of incubation in 3% acetic acid (3 minutes), followed by staining in Alcian Blue solution (30 minutes), a brief rinse in 3% acetic acid and distilled water, and counterstaining with Nuclear Fast Red (3 minutes). Sirius Red staining was performed manually by applying Picro-Sirius Red solution for 1 hour, followed by two 1-minute washes in 30% acetic acid. Dehydration and clearing steps for both stainings were identical: slides were immersed in ascending ethanol concentrations (70%, 80%, 100%; 1 min each), cleared in xylene (2 × 1 minute), and mounted with EUKITT® (Sigma-Aldrich, 03989-500ML) and a coverslip.

Immunohistochemistry

Paraffin-embedded sections were incubated at 65 °C for 1 h, followed by deparaffinisation in xylene and rehydration through graded ethanol. Antigen retrieval was performed by steaming slides in preheated buffer for 20 minutes, followed by cooling to room temperature. Slides were washed with TBST (Tris-buffered saline with Tween20® detergent, 0.05M Tris, 0.9% sodium chloride, pH 8.4) and endogenous peroxidase activity was quenched with Bloxall (Vector Laboratories, SP-6000-100) for 30 minutes in the dark. Sections were blocked with horse serum (Vector Laboratories, S-2012-50) for 1 hour in a humid chamber and then incubated with primary antibody overnight at 4 °C in a humidified chamber. The following day, slides were washed with TBST and incubated with ImmPRESS HRP polymer secondary antibody (Vector Laboratories, MP-7401 or MP-7402) for 30 minutes. After washing (2×10 minutes, TBST), detection was performed using ImmPACT® DAB Substrate, Peroxidase HRP (Vector Laboratories, SK-4105; 30 µL DAB in 1 mL buffer) for 1 minute. Slides were rinsed in distilled water, counterstained with hematoxylin and washed under running tap water. Sections were dehydrated through ethanol, cleared in xylene and mounted with Rotimount under coverslips. Anti-Cytokeratin 19 (Abcam, ab133496) antibody was diluted 1:500 and Anti-alpha smooth muscle actin (Abcam, ab5694) antibody was diluted 1:100 in Dako (Agilent, S0809), while Basp-1 (Abcam, ab312324) was diluted 1:8000. For mouse primary antibody, the Mouse-on-Mouse (M.O.M.) ImmPRESS HRP Polymer Kit (Vector Laboratories, MP-2400) was used. After quenching, sections were blocked with M.O.M. IgG blocking reagent and incubated with 2.5% normal horse serum (NHS). Primary antibody MUC5AC Monoclonal Antibody (Invitrogen, MA5-12178) was diluted in NHS at a dilution of 1:100 and applied for 30 min. M.O.M. ImmPRESS reagent was applied, followed by final washes.

Imaging and analysis

Whole-slide imaging was performed using the Axio Scan.Z1 Slide Scanner (Zeiss) using the 20X objective. Image acquisition was conducted using the ZEN 3.7 software (Zeiss). All tissue sections, including those stained with H&E, Alcian blue, Sirius red and IHC staining of Cytokeratin-19, Basp-1, Muc5ac and alpha-SMA, were scanned under identical settings to ensure consistency across samples. Subsequent image analysis was carried out using QuPath (version 0.5.0), an open-source software for bioimage analysis with integrated machine learning capabilities. Analysis workflows were established in accordance with publicly available guidelines and tutorials provided on the QuPath website

(https://qupath.github.io/). For Alcian Blue and Sirius Red stainings the analysis focused on quantifying the positively stained areas. These images were analysed using the “Brightfield (other)” setting in QuPath. Three tissue categories were defined: “positive” for intensely blue (Alcian Blue) or red (Sirius Red) stained regions, “other” for less intensely stained areas, and non-stained regions, which were excluded from analysis. Pixel classification, including Muc5ac and alpha-SMA quantification, was performed using a supervised learning approach. Once the classifier was adequately trained, it was applied to the entire tissue section, and the proportion of positively stained area was calculated. Results from all QuPath analysis were exported for further statistical processing. H&E-stained sections were independently evaluated by two experienced pathologists for evidence of tissue remodelling, with results expressed as percentage estimates. These level of remodelling assessments were cross-validated using QuPath-based quantitative analysis of H&E staining. Additionally, all sections were examined for presence of ADM (acinar-to-ductal metaplasia), PanIN LG (pancreatic intraepithelial neoplasia, low-grade) and HG (high-grade), AFL (atypic flat lesion) and pancreatic ductal adenocarcinoma (PDAC) independently by two experienced pathologists.

Each genotype and time point was analysed with at least four animals or more. When there was more than one section per animal in the H&E staining, the average was calculated and used for further analysis. Statistical significance was determined by the unpaired, two-tailed t-test. The following nomenclature was used to indicate significance: not significant (ns) for $P > 0.05$, * for $P \leq 0.05$, ** for $P \leq 0.01$, *** for $P \leq 0.001$, **** for $P \leq 0.0001$. Statistical analysis of all quantified data was performed using *GraphPad Prism* v10 (GraphPad Software Inc.).

Isolation and transdifferentiation of murine acinar cells

After euthanasia, the mouse pancreas was removed and washed with ice cold 1X PBS (Phosphate Buffered Saline, Gibco™, 10010023). The pancreas was cut into small pieces and digested using Collagenase P at a concentration ranging from 0.5mg/mL to 2mg/mL. Digestion was carried out at 37°C for 20-30 minutes. The digested tissue was pipetted up and down to resolve the cells out of the tissue pieces and passed through a 100µm cell strainer. The cell strainer was rinsed with RPMI medium supplemented with 5% Fetal Calf Serum (FCS). The acini-enriched suspension was centrifuged at 300 x g for 5 minutes. The resulting pellet was gently washed twice with Hank’s Balanced Salt Solution (HBSS) without phenol red and resuspended in 2D culture medium composed of Waymouth’s MB 752/1

(Gibco™, 31220023), 10% FCS, 1% penicillin-streptomycin, 0.1mg/ml collagenase from soybean (Gibco™, 17075029) , 5mM HEPES (Gibco™, 15630106), 0.13% sodium bicarbonate and 0.002 mg/ml dexamethasone (Sigma-Aldrich, D4902). The acinar enriched suspension was divided in two parts where one portion was treated with 100 pM caerulein (Sigma-Aldrich, C9026) overnight. The next day, the acini were recovered and washed with HBSS and resuspended in 3D culture medium composed of Waymouth's MB 752/1, 20% FCS, 2% penicillin-streptomycin, 0.2mg/ml collagenase from soybean, 10mM HEPES, 0.26% sodium bicarbonate and 0.004 mg/ml dexamethasone. The cell suspension was mixed in a ratio of 1:1 with neutralized collagen and seeded on collagen-coated wells. After the collagen was set, 2D culture medium with or without 50ng/ml TGF-α (R&D Systems, 239-A-100) was added and incubated at 37°C, 5% CO2. Transdifferentiation was observed over a period of 4 days by live-cell imaging using Incucyte (Sartorius).

An artificial neural network-based pixel classifier detecting ADM areas was trained on isolations not included in the analysis in QuPath (v0.6.0), using default settings and local normalization of mean & variance. Once the classifier was adequately trained, it was applied to the live-cell images of KC-Cpa1 and KC isolations. To exclude false positives including acini and detritus, we defined the baseline of the analysis as the mean detected area of 6 hours to 1 day. ADM areas at each individual time point were then normalized to the mean area of early ADM 1 day 6 hours to 2 days 6 hours, thus capturing the increase of ADM area independent of primary acini density.

Cpa1^{N256K} retroviral overexpression in 266-6 cells

266-6 cells (RRID: CVCL_3481) were obtained from Prof. I. Rooman (VUB, Brussels, Belgium) who obtained them from ATCC (CRL-2151), and cultured in DMEM supplemented with 10% FCS. Myc-tagged murine *Cpa1* cDNA (Gene ID: 109697) and corresponding *Cpa1*^{N256K} cDNA were synthetized by VectorBuilder Inc. in a MMLV (mouse moloney leucemia virus) based ectopic retroviral vector. Insert sequences were checked using enzymatic digestion and Sanger sequencing. Retroviral particles were produced using the ecotropic packaging cell line HEK293T Platinum-E (Plat-E, Cell Biolabs Inc., RV-101, RRID: CVCL_B488) maintained in DMEM supplemented with 10% FCS, 1µg/mL puromycin, and 10µg/mL blasticidin. For virus production, 1 × 10^6 HEK293T Platinum E cells were seeded in each well of a 6-well plate in antibiotic-free DMEM containing 10% FCS. Cells were transfected 3–5 hours post-seeding with 5µg of plasmid DNA per well. Transfection mixes were prepared by combining DNA with

sterile H_2O and 2X HBS followed by the addition of 2.5M $CaCl_2$. After 20 minutes of incubation at room temperature, the mix was added dropwise to the cells. Sixteen hours post-transfection, the medium was replaced with fresh DMEM containing 10% FCS without antibiotics. Viral supernatants were collected 48 hours after transfection, filtered through a 0.45µm membrane, and either used immediately or stored at -80°C. 266-6 were seeded at 20 - 30% confluency one day prior to transduction. For infection, the medium was replaced with fresh culture medium supplemented with 2µg/mL polybrene, and filtered viral supernatant was added at a 1:2 dilution. After 24 hours, the medium was replaced with selection medium containing puromycin at the appropriate concentration. Transduction efficiency was monitored microscopically, and stable cell populations were expanded once confluency reached 80–95%. Cells were collected for RNAseq analysis after culturing for 24 hours. RNA for RNAseq experiments was isolated using RNeasy Mini Kit (Qiagen, 74106).

RNA isolation

For bulk RNAseq experiments, 2-, 4- and 8-week-old mice and mice from the ageing cohort were sacrificed via isoflurane overdose (Vetflurane, Virbac) followed by cervical dislocation to ensure euthanasia. Pancreata were prepared snap-frozen in liquid nitrogen and stored at -80°C. Total RNA was isolated from pancreatic tissue using a combination of QIAzol lysis reagent (Qiagen, 79306) and chloroform-based (Serva, 39553.01) phase separation, followed by purification with the RNeasy Mini Kit (Qiagen, 74106), according to the manufacturer's protocol. Approximately 2–3 mm^3 of snap-frozen tissue was transferred into 200 µL of ice-cold QIAzol containing 1% β-mercaptoethanol (Sigma Aldrich, M6250). Samples were immediately frozen in liquid nitrogen and stored at –80°C until further processing. For homogenisation, a sterile, RNase-free stainless-steel bead was added, and 1.3 mL of additional ice-cold QIAzol was added while maintaining the samples on dry ice to prevent thawing. Tissue homogenisation was performed using a pre-cooled Qiagen TissueLyser II at 30 Hz for 20 minutes at 4°C. Lysates were centrifuged at 13,000 rcf for 5 minutes at 4°C, and the supernatant was carefully transferred to a fresh tube, avoiding residual tissue fragments. Phase separation was performed by adding 300 µL of ice-cold chloroform, followed by vigorous vortexing for 10 seconds. After centrifugation at 13,000 rcf for 5 minutes at 4°C, the upper aqueous phase (~600 µL) was carefully collected, avoiding contamination from the interphase, and mixed with an equal volume of isopropanol (Fisher Scientific, BP2618-500). RNA purification was completed using the RNeasy Mini Kit, including on-column DNase I digestion (Macherey-Nagel, 740963) to eliminate residual genomic DNA.

RNA sequencing and analysis

For mRNA-seq libraries, polyA-RNA was enriched using oligo(dT) beads. Generation of libraries and sequencing were performed by Novogene (Cambridge, UK, or Munich, Germany) on an Illumina NovaSeq 6000 platform for mouse and on an Illumina NovaSeq X Plus platform for 266-6 cell line samples. Adapter sequences as well as low quality read ends were clipped off using Cutadapt[9] (v2.8). The processed sequencing reads were aligned to the mouse reference genome (UCSC mm10) using HiSat2[10]. Samtools[11] was used to extract primary alignments and to index the resulting BAM files. FeatureCounts[12] (v2.0.0) was used for summarising gene-mapped reads. ENSEMBL GRCm38 v.100[13] was used as annotation basis. Differential gene expression was determined using the R package edgeR[14] (v4.2.1) utilising trimmed mean of M-values[15] normalisation. For mouse experiment data, the exact Test function was applied to obtain genes differentially expressed between any two conditions. A false discovery rate (FDR) value below 0.05 was considered as threshold for significant differential gene expression. FPKM normalised expression values were obtained for further analyses. For 266-6 cell line experiment data, differentially expressed genes were determined by fitting a generalized linear model using the glmFit() function of edgeR, blocking for replicate batch. Gene set enrichment analysis (GSEA) was performed using the R-package clusterProfiler[16] (v4.12.16) and murine homologs of MSigDB[17] (v5.51) gene sets for mouse data and MSigDB gene sets (v2023.1) for 266-6 data utilising the fgsea algorithm. The exponent parameter was set to 0 for unweighted analyses of log2 fold change sorted gene lists from RNA-seq data.

Preprocessing scRNA-seq

FASTQ files were aligned to the reference genome GRCm39 (2024-A) using Cell Ranger (10x Genomics Inc., v9.0.0). Count matrices were then merged and preprocessed in RStudio[18] (R, v4.3.2[19]), using Seurat[20] (v5.0.3). Doublets were detected using scDblFinder (v1.16.0). Low-quality cells were filtered based on cut-offs (> 200 detectable genes, > 1,000 unique molecular identifiers, < 20% mitochondrial transcripts) defined in a recent benchmarking publication[21]. Normalisation was performed via SCTransform[22] (variable features n = 5,000, v2 regularisation), regressing out cell cycle scores and the percentage of mitochondrial genes expressed. Dimensionality was reduced using principal component analysis (PCA). UMAP (Uniform Manifold Approximation and Projection)[23] was applied to visualise cells in two-dimensional plots. Cells were clustered using Seurat's implementation of the

Louvain algorithm[23,24]. Clusters were assessed for feature counts, mitochondrial genes and acinar markers to exclude clusters of ambient acinar RNA from downstream analyses, followed by renormalization and reclustering. Clusters were then annotated using our annotation strategy (online supplemental table 1), based on literature and recent single-cell studies.

Single-cell analysis

Pseudotime trajectories were inferred using Monocle3[25] (v1.3.4). Cell states along the inferred trajectories were interpolated using cellAlign[26] (v0.1.0). Pseudobulk profiles of individual cell types were calculated by aggregating normalised gene expression across the entire measured transcriptome. These pseudobulk profiles were then subjected to Pearson correlation. Cell-cell interactions were predicted using CellChat[27] (v2.1.2). Gene set enrichment analysis (GSEA) of murine KEGG pathways was performed using Seurat's implementation of the enrichR[28] (v3.2) database and clusterProfiler[16] (v4.10.1). Differentially expressed genes (DEGs) were determined using global Wilcoxon rank-sum test statistics across genotypes and clusters. DEGs along the inferred KC-Cpa1 trajectory were determined using Monocle3's implementation of the Moran's I test statistic.

Spatial transcriptomics experiments

Pancreata of 8-week-old male Cre and Cpa1 mice were isolated. Mice were euthanised by cervical dislocation, pancreata immediately isolated, rinsed with cold PBS buffer, and processed as described in the histology section. Paraffin blocks were stored at 4°C until further processing. Tissue blocks were incubated on ice bath 20 minutes before sectioning. For each specimen, two sections at 10 µm thickness were cut, and RNA was isolated using Deparaffinisation Solution (Qiagen GmbH, 19093), and RNeasy®FFPE Kit for RNA Extraction (Qiagen GmbH, 73504) according to the manufacturer's protocol. RNA quality was assessed by measuring the percentage of total RNA fragments greater than 200 nucleotides (DV200) with the 2100 Bioanalyzer Instrument using the Agilent RNA 6000 Nano Kit (Agilent Technologies, Inc., 5067-1511). Cre DV200 was 83%, Cpa1 DV200 48%; 10x Genomics recommended minimum requirement 30%. Next, tissue sections at 5 µm thickness were prepared and placed on poly-L-lysine-coated glass slides (Sigma-Aldrich Co., P0425-72EA) and dried for 30 minutes on a heating plate at 42°C, followed by subjecting to a 42°C dry-incubation in an oven for three hours. Subsequently, the sections were stored at room temperature in a desiccator for 24 hours. Library preparation process was conducted in accordance with the manufacturer's protocols (10x Genomics Inc.; Visium HD FFPE

Tissue Preparation Handbook, CG000684 and Spatial Gene Expression Reagent Kits User Guide, CG000685), and reagents (Visium HD Spatial Gene Expression Reagent Kit – small, 1000668; and Visium HD Mouse Transcriptome Probes v2 – small, 1000667). In brief, the sections were deparaffinised and H&E stained to assess tissue morphology. For deparaffinisation tissue slides were incubated for two hours at 60°C in an oven, cooled for five minutes at room temperature and deparaffinised by immersing slides twice for 10 minutes in xylene (Sigma-Aldrich, 214736). Next, slides were rehydrated in an ethanol (Fisher Scientific GmbH, 17740239) series consisting of 2 × 100%, 2 × 96%, and 1 × 70% ethanol, with each step lasting three minutes, following incubation in distilled water for 20 seconds. Deparaffinised and rehydrated slides were stained with 1 mL of Gill II hematoxylin solution (Sigma-Aldrich, GHS232) and incubated at room temperature for 30 seconds. Excessive staining solution was removed by immersing the slides in three water beakers. 1 mL of the bluing buffer (Agilent Technologies, Inc., CS70230-2) was added, and slides were incubated for one minute prior to being immersed in a water beaker. Subsequent staining was performed using alcoholic eosin (Sigma-Aldrich, HT110116) by applying 1 mL of eosin for one minute and washing out by immersing the slides in two water beakers. Finally, slides were cover slipped using 85% glycerol (Thermo Scientific Chemicals, 327255000) mounting medium. For imaging Axio Scan Z.1 slide scanner (Carl Zeiss AG) was used, and the area of interest (AOI) was determined. Following the removal of the coverslips, tissue slides were placed in the tissue cassettes (10x Genomics Inc., Visium Tissue Slide Cassette S3, 1000684), and destained to remove residual hematoxylin and eosin. Subsequent to the initial destaining step, samples underwent decrosslinking to enhance the accessibility of probes to target RNA molecules. Then, hybridisation was performed using mouse transcriptome specific probes. In the subsequent phase – probe ligation – a ligase was incorporated, resulting in the formation of a ligation product. The cassettes were then maintained at a temperature of 4°C. Visium HD slide (10x Genomics Inc., Visium HD Slide, 1000670) was thawed and prepared prior to the execution of the Visium CytAssist device. Further steps, including probe release and capture, are facilitated within the Visium CytAssist device. Subsequent to being released from the tissue, the ligation products were captured on the Visium slide and extended by the addition of the spatial barcode. The spatially-barcoded ligation products were amplified, followed by SPRIselect cleanup (SPRIselect DNA Size Selection Reagent, Beckman Coulter Inc., B23317). Next, quantitative polymerase chain reaction (qPCR) was performed to establish the sample index PCR cycle number for gene expression libraries. Following amplification, the material was subjected to indexing via sample index PCR, thereby generating library molecules. Subsequent to the cDNA amplification, the

libraries were purified using SPRIselect and qualitatively assessed with the 2100 Bioanalyzer Instrument using the High Sensitivity DNA Kit (Agilent Technologies Inc., 5067-4626).

The final libraries were sequenced by Novogene (Munich, Germany) on an Illumina NovaSeq X Plus Series platform in paired-end mode (PE150). Raw data was obtained in FASTQ format.

Spatial transcriptomics analysis

Reads and images were processed using Space Ranger (v3.1.3). Subsequent analyses were performed in RStudio[18] (R, v4.3.2[19]), using Seurat[20] (v5.0.3). Spatial deconvolution of 16 µm bins was performed using robust cell type decomposition (RCTD) (R package spacexr, v2.2.1[29]), utilizing the single-cell data of 8-week-old Cre and Cpa1 mice as a reference. Analyses were run in doublet detection mode "doublet", and non-epithelial bins were termed either "immune" (granulocyte, T & NK cell, macrophage & dendritic cell, B cell) or "connective" (lymphatic endothelial, pericyte, red blood cell, endothelial, fibroblast, schwann, mesothelial) following deconvolution. Tissue domains were predicted using BANKSY[30] (Building Aggregates with a Neighborhood Kernel and Spatial Yardstick) (v1.5.3). 16 µm binned count matrices of both samples were log-normalized, 2,000 variable features were determined for each sample and BANKSY neighborhood feature matrices were calculated based on the union Haematoxylin of highly variable genes. BANKSY PCA embeddings of both samples were integrated via Harmony[31] (v1.2.3) and bins were clustered using Seurat's implementation of the Louvain algorithm[24]. UMAP (Uniform Manifold Approximation and Projection)[23] was applied to visualise tissue domains in two-dimensional plots.

REFERENCES

1. Hegyi E, Sahin-Tóth M. Human CPA1 mutation causes digestive enzyme misfolding and chronic pancreatitis in mice. Gut 2019;68:301–312.

2. Hingorani SR, Petricoin EF, Maitra A, et al. Preinvasive and invasive ductal pancreatic cancer and its early detection in the mouse. Cancer Cell 2003;4:437–450.

3. Kawaguchi Y, Cooper B, Gannon M, et al. The role of the transcriptional regulator Ptf1a in converting intestinal to pancreatic progenitors. Nat Genet 2002;32:128–134.

4. Natale CA, Li J, Pitarresi JR, et al. Pharmacologic Activation of the G Protein-Coupled Estrogen Receptor Inhibits Pancreatic Ductal Adenocarcinoma. Cell Mol Gastroenterol Hepatol 2020;10:868-880.e1.

5. Carrière C, Young AL, Gunn JR, et al. Acute pancreatitis markedly accelerates pancreatic cancer progression in mice expressing oncogenic Kras. Biochem Biophys Res Commun 2009;382:561–565.

6. Varghese M, Griffin C, Abrishami S, et al. Sex hormones regulate metainflammation in diet-induced obesity in mice. J Biol Chem 2021;297:101229.

7. Chang H-H, Moro A, Takakura K, et al. Incidence of pancreatic cancer is dramatically increased by a high fat, high calorie diet in KrasG12D mice. PloS One 2017;12:e0184455.

8. Lee W, Yang E, Curley JP. Foraging dynamics are associated with social status and context in mouse social hierarchies. PeerJ 2018;6:e5617.

9. Martin M. Cutadapt removes adapter sequences from high-throughput sequencing reads. EMBnet.journal 2011;17:10–12.

10. Kim D, Langmead B, Salzberg SL. HISAT: a fast spliced aligner with low memory requirements. Nat Methods 2015;12:357–360.

11. Li H, Handsaker B, Wysoker A, et al. The Sequence Alignment/Map format and SAMtools. Bioinforma Oxf Engl 2009;25:2078–2079.

12. Liao Y, Smyth GK, Shi W. featureCounts: an efficient general purpose program for assigning sequence reads to genomic features. Bioinforma Oxf Engl 2014;30:923–930.

13. Aken BL, Achuthan P, Akanni W, et al. Ensembl 2017. Nucleic Acids Res 2017;45:D635–D642.

14. Robinson MD, McCarthy DJ, Smyth GK. edgeR: a Bioconductor package for differential expression analysis of digital gene expression data. Bioinformatics 2010;26:139–140.

15. Robinson MD, Oshlack A. A scaling normalization method for differential expression analysis of RNA-seq data. Genome Biol 2010;11:R25.

16. Yu G, Wang L-G, Han Y, et al. clusterProfiler: an R Package for Comparing Biological Themes Among Gene Clusters. OMICS J Integr Biol 2012;16:284–287.

17. Liberzon A, Subramanian A, Pinchback R, et al. Molecular signatures database (MSigDB) 3.0. Bioinformatics 2011;27:1739–1740.

18. Posit team. RStudio: Integrated Development Environment for R. 2024. Available at: http://www.posit.co/.

19. R Core Team. R: A Language and Environment for Statistical Computing. 2023. Available at: https://www.R-project.org/.

20. Hao Y, Stuart T, Kowalski MH, et al. Dictionary learning for integrative, multimodal and scalable single-cell analysis. Nat Biotechnol 2024;42:293–304.

21. Aney KJ, Jeong W-J, Vallejo AF, et al. Novel Approach for Pancreas Transcriptomics Reveals the Cellular Landscape in Homeostasis and Acute Pancreatitis. Gastroenterology 2024;0. Available at: https://www.gastrojournal.org/article/S0016-5085(24)00130-6/fulltext#secsectitle0105 [Accessed March 18, 2024].

22. Hafemeister C, Satija R. Normalization and variance stabilization of single-cell RNA-seq data using regularized negative binomial regression. Genome Biol 2019;20:296.

23. Becht E, McInnes L, Healy J, et al. Dimensionality reduction for visualizing single-cell data using UMAP. Nat Biotechnol 2018.

24. Blondel VD, Guillaume J-L, Lambiotte R, et al. Fast unfolding of communities in large networks. J Stat Mech Theory Exp 2008;2008:P10008.

25. Cao J, Spielmann M, Qiu X, et al. The single-cell transcriptional landscape of mammalian organogenesis. Nature 2019;566:496–502.

26. Alpert A, Moore LS, Dubovik T, et al. Alignment of single-cell trajectories to compare cellular expression dynamics. Nat Methods 2018;15:267–270.

27. Jin S, Plikus MV, Nie Q. CellChat for systematic analysis of cell–cell communication from single-cell transcriptomics. Nat Protoc 2025;20:180–219.

28. Kuleshov MV, Jones MR, Rouillard AD, et al. Enrichr: a comprehensive gene set enrichment analysis web server 2016 update. Nucleic Acids Res 2016;44:W90–W97.

29. Cable DM, Murray E, Zou LS, et al. Robust decomposition of cell type mixtures in spatial transcriptomics. Nat Biotechnol 2022;40:517–526.

30. Singhal V, Chou N, Lee J, et al. BANKSY unifies cell typing and tissue domain segmentation for scalable spatial omics data analysis. Nat Genet 2024;56:431–441.

31. Korsunsky I, Millard N, Fan J, et al. Fast, sensitive and accurate integration of single-cell data with Harmony. Nat Methods 2019;16:1289–1296.
